# The maternal to zygotic transition regulates genome-wide heterochromatin establishment in the zebrafish embryo

**DOI:** 10.1038/s41467-019-09582-3

**Published:** 2019-04-04

**Authors:** Kathrin Laue, Srivarsha Rajshekar, Abigail J. Courtney, Zachary A. Lewis, Mary G. Goll

**Affiliations:** 1 Department of Genetics, University of Georgia, Athens, GA 30602 USA; 2 Developmental Biology Program, Memorial Sloan Kettering Cancer Center, New York, NY 10065 USA; 3 Program in Biochemistry and Structural Biology, Cell and Developmental Biology, and Molecular Biology, Weill Cornell Graduate School of Medical Sciences, Cornell University, New York, NY 10065 USA; 4 Department of Microbiology, University of Georgia, Athens, GA 30602 USA; 5 Present Address: Department of Genetics, University of Georgia, Athens, GA 30602 USA

## Abstract

The segregation of eukaryotic genomes into euchromatin and heterochromatin represents a fundamental and poorly understood process. Here, we demonstrate that genome-wide establishment of heterochromatin is triggered by the maternal to zygotic transition (MZT) during zebrafish embryogenesis. We find that prior to MZT, zebrafish lack hallmarks of heterochromatin including histone H3 lysine 9 trimethylation (H3K9me3) and condensed chromatin ultrastructure. Global establishment of heterochromatic features occurs following MZT and requires both activation of the zygotic genome and degradation of maternally deposited RNA. Mechanistically, we demonstrate that zygotic transcription of the micro RNA miR-430 promotes degradation of maternal RNA encoding the chromatin remodeling protein Smarca2, and that clearance of Smarca2 is required for global heterochromatin establishment in the early embryo. Our results identify MZT as a key developmental regulator of heterochromatin establishment during vertebrate embryogenesis and uncover functions for Smarca2 in protecting the embryonic genome against heterochromatinization.

## Abstract (web summary)

Eukaryotic genomes are segregated into euchromatin and heterochromatin. Here the authors show that heterochromatin establishment during zebrafish embryo development is controlled by zygotic transcription of miR-430 and subsequent degradation of maternal transcripts encoding the chromatin remodeling protein Smarca2.

## Introduction

The segregation of eukaryotic genomes into regions of euchromatin and heterochromatin is fundamental to genome organization. At the molecular level, these domains are distinguished by different levels of chromatin compaction and unique sets of histone modifications. Highly condensed, constitutive heterochromatin is marked by trimethylation of histone H3 lysine 9 (H3K9me3) and is found predominately at repetitive sequences across the genome. Heterochromatin formation at these sequences promotes transcriptional repression, as well as genome stability, and depletion of H3K9me3 marked heterochromatin severely impairs viability in mice, flies, and zebrafish^1–3^.

Although the timing and extent varies between species, developmental reprogramming of H3K9me3 marked heterochromatin has been noted in diverse metazoa, including mammals, flies, and *Caenorhabditis elegans*^4–9^. Heterochromatin establishment coincides with diminishing cellular plasticity in these species, and heterochromatin formation also represents a major barrier to cellular reprogramming^10,11^. The inverse relationship between chromatin compaction and developmental potential suggests that the timing of heterochromatin formation must be tightly regulated during development^11,12^. However, the mechanisms that control global establishment of heterochromatic states remain largely unknown, especially in the context of vertebrate embryogenesis.

In this study, we set out to characterize heterochromatin regulation in the context of early zebrafish development. We find that the zebrafish embryo undergoes at least 10 rounds of cell division in the absence of condensed chromatin ultrastructure. Global establishment of H3K9me3 and chromatin compaction is first noted following the maternal to zygotic transition (MZT) and the establishment of heterochromatin is dependent on this transition. At the molecular level, we show that zygotic transcription of the microRNA miR-430 promotes degradation of maternal RNA encoding the ATP-dependent chromatin remodeler Smarca2, and that clearance of Smarca2 is required for H3K9me3 establishment and chromatin compaction following MZT. Our study identifies MZT as a key regulator of de novo heterochromatin establishment in the early zebrafish embryo and reveals functions for Smarca2 in antagonizing the de novo establishment of heterochromatin during early vertebrate embryogenesis.

## Results

### Chromatin of the early zebrafish embryo lacks H3K9me3

**Fig. 1:**
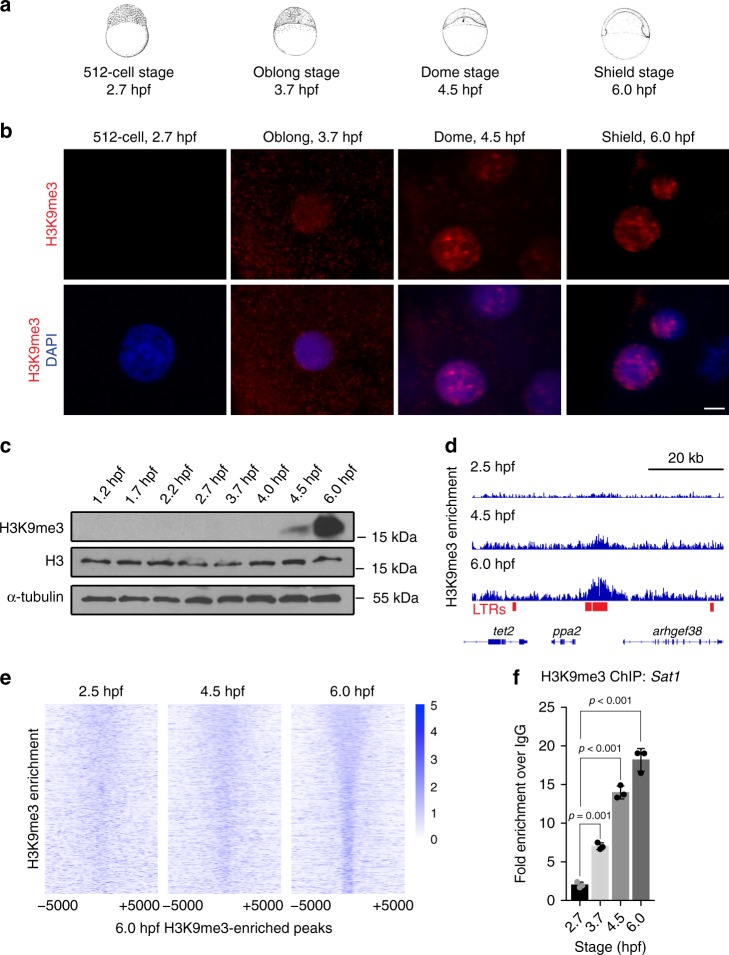
Chromatin of the early zebrafish embryo lacks H3K9me3. **a** Developmental stages used for heterochromatin analysis. Zebrafish embryonic stage schematics are reproduced from Kimmel et al.^22^ with permission from John Wiley & Sons Inc. **b** Representative images showing H3K9me3 immunofluorescent antibody labeling of nuclei from fixed embryos at 2.7, 3.7, 4.5, and 6 h post fertilization (hpf). Images are representative of three independent experiments with at lease 15 embryos observed at each time point in each experiment. Top panels show H3K9me3 labeling (red). Bottom panels show H3K9me3 (red) overlaid with 4′,6-diamidino-2-phenylindole (DAPI) (blue). All images were taken under identical conditions, the scale bar represents 1 μm. **c** Western blots for H3K9me3, histone H3, and α-tubulin. For each time point, total protein extracts were isolated from 20 embryos, and one-third of the protein extract for each sample was loaded for western blot. **d**, **e** H3K9me3 enrichment measured by chromatin immunoprecipitation with sequencing (ChIP-seq) at 2.5, 4.5, and 6 hpf. **d** Screen shot of H3K9me3 enrichment at an LTR transposon in a representative genomic region. **e** Heatmap depicting H3K9me3 enrichment across all 17,621 H3K9me3 peaks identified in 6 hpf embryos. Signals are centered on peak centers and include ±5000 bp. **f** H3K9me3 enrichment at Satellite-1 (Sat1) pericentromeric repeats at 2.7, 3.7, 4.5, and 6 hpf, measured by ChIP and presented as fold enrichment over an immunoglobulin G (IgG)-only control. *P* values were calculated by ordinary one-way analysis of variance (ANOVA), with corrections for multiple testing. Error bars indicate the standard error of the mean (SEM). Source data for panel c are provided as a source data file

In order to characterize the heterochromatic compartment of the early zebrafish genome, we collected embryos during the period between early blastula (512-cell, 2.7 h post fertilization (hpf)), and mid gastrula (shield, 6 hpf) stages and assessed embryonic nuclei for H3K9me3-rich foci (Fig. 1a). These H3K9me3 marked foci, termed chromocenters, serve as a classical cytological marker of constitutive heterochromatin, and are readily detected in mouse embryos as early as the two-cell stage^3^. We observed that while chromocenters were obvious in nuclei of shield stage zebrafish embryos (6 hpf), they were undetectable in nuclei at the 512-cell stage (2.7 hpf) (Fig. 1b). Faint, diffuse nuclear H3K9me3 labeling was first observed in embryos at oblong stage (3.7 hpf), and emergent foci were noted by dome stage (4.5 hpf) (Fig. 1b). H3K9me3 was also undetectable by western blot in bulk histones isolated at 4.0 hpf and earlier time points, but was abundant in dome (4.5 hpf) and shield stage (6 hpf) embryos (Fig. 1c, Supplementary Fig. 1a). Chromatin immunoprecipitation with sequencing (ChIP-seq) supported these findings. At 6 hpf, regions enriched for H3K9me3 were detected across the genome, with ~96% of the called H3K9me3 peaks overlapping annotated repeats (Fig. 1d, e, Supplementary Fig. 1b–d). At 4.5 hpf, low amplitude peaks were observed within a subset of regions showing enrichment at 6 hpf. However, little signal was detected within these regions at 2.5 hpf. Enrichment of H3K9me3 at 4.5 and 6 hpf was observed at DNA, LINE, LTR, and SINE transposons, with the most peaks detected in LTR transposons (Supplementary Fig. 1e). The small fraction of peaks not corresponding to repetitive elements were found mainly in intergenic regions. Similar temporal enrichment of H3K9me3 was observed when H3K9me3 enrichment was directly examined at pericentromeric Satellite-1 (Sat1) repeats (Fig. 1f). These findings suggest that the early zebrafish embryo undergoes many rounds of cell division in an environment that is globally depleted for H3K9me3 marked heterochromatin, and that a major wave of H3K9me3 establishment initiates around 3.7 hpf.

### Early embryos lack condensed chromatin ultrastructure

**Fig. 2:**
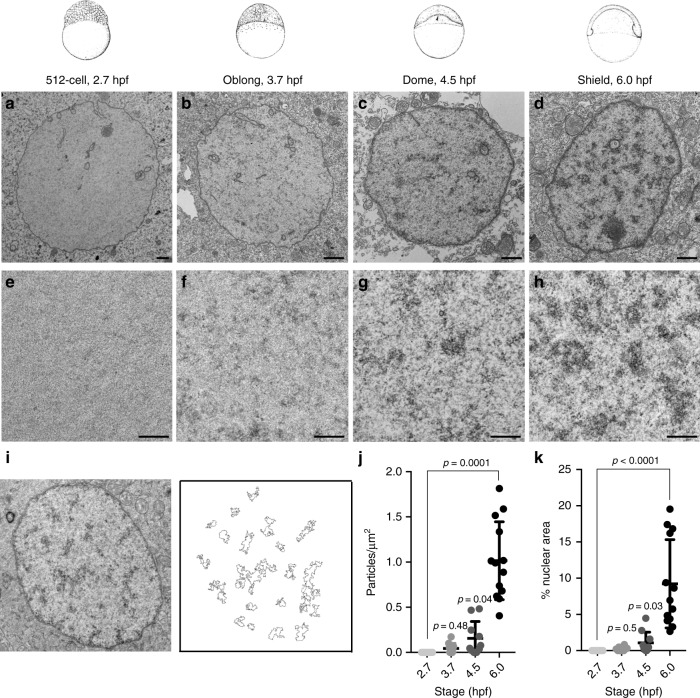
The early zebrafish embryo lacks condensed chromatin ultrastructure. Zebrafish embryonic stage schematics are reproduced from Kimmel et al.^22^ with permission from John Wiley & Sons Inc. **a**–**h** Transmission electron micrographs (TEMs) of representative nuclei from embryos at 2.7, 3.7, 4.5, and 6 h post fertilization (hpf). Images of representative nuclei at specified time points. **e**–**h** Higher magnification images (×20,000) of nuclear interior at specified time points. All scale bars (**a**–**h**) indicate 1 μm. **i** Representative image illustrating particle selection. **j** Quantification of the number of particles per nuclear μm^2^ in TEM images at 2.7, 3.7, 4.5, and 6 hpf. **k** Quantification of the percent nuclear area covered by particles in TEM images at 2.7, 3.7, 4.5, and 6 hpf. Nuclei from three embryos were assessed per time point, and electron-dense aggregates were quantified in four to six representative nuclei from each embryo. Each point represents data from one nucleus. *P* values were calculated using the Kruskal–Wallis test and corrected for multiple comparisons, error bars indicate standard deviation (SD)

To characterize embryonic heterochromatin at the ultrastructure level, we next turned to transmission electron microscopy (TEM) (Fig. 2a–h and Supplementary Fig. 2a). As expected, electron-dense aggregates indicative of condensed chromatin ultrastructure were clearly visible within nuclei from shield stage embryos (6 hpf) (Fig. 2d, h). However, at the 512-cell stage (2.7 hpf), these aggregates were undetectable (Fig. 2a, e). Aggregates were first noted in some embryonic nuclei at the oblong stage (3.7 hpf), and appeared more common by the dome stage (4.5 hpf) (Fig. 2b, c, f, g). To quantify these observations, maximum entropy thresholding was used to define and count the number of electron-dense particles relative to nuclear area in individual cells from three embryos per time point (Fig. 2i)^13^. At the 512-cell stage (2.7 hpf), electron-dense aggregates exceeding a particle size of 0.03 μm^2^ were not detected in embryonic nuclei, suggesting a lack of condensed ultrastructure. Significant increases in the number of nuclear aggregates per μm^2^ and the percent nuclear area covered by aggregates were first noted at the dome stage (4.5 hpf), and increased 7- and 9-fold by shield stage (6 hpf) (Fig. 2j, k). Consistent with these increases in chromatin compaction, we observed decreased expression of transcripts derived from repetitive elements between 4 and 6 hpf (Supplementary Fig. 2b, e). These data indicate that the genome of the early zebrafish is packaged in an atypically decondensed chromatin state, and that embryonic chromatin undergoes a profound reorganization involving the establishment of condensed chromatin ultrastructure between 3.7 and 6 hpf.

### Blocking zygotic transcription impairs heterochromatin

**Fig. 3:**
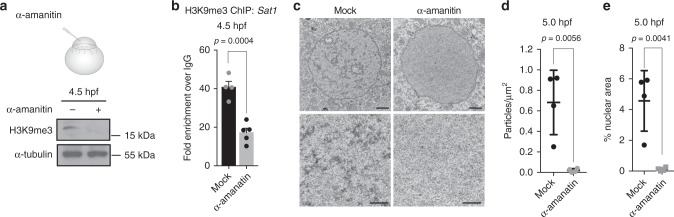
Blocking zygotic transcription impairs heterochromatin. **a** Western blot for H3K9me3 (top) and α-tubulin (bottom) using protein extracted from embryos that were either mock injected (−) or injected with 0.2 ng of α-amanitin (+) at the one-cell stage. Protein was collected for analysis at 4.5 h post fertilization (hpf). **b** Chromatin immunoprecipitation with sequencing (ChIP) for H3K9me3 enrichment at pericentromeric Satellite-1 (Sat1) repeats in wild-type and α-amanatin-injected embryos. Embryos were collected for analysis at 4.5 hpf. *P* values were calculated using the Student’s *t* test; error bars indicate SEM. **c** Transmission electron microscopy (TEM) images demonstrating a lack of condensed chromatin ultrastructure in 5.0 hpf embryos that were injected with α-amanatin at the one-cell stage. Bottom panels represent higher magnification images (×20,000) of nuclear interior in mock- and α-amanatin-injected embryos at specified time points. Scale bars indicate 1 μm. **d**, **e** Quantification of the number of electron-dense aggregates per nuclear μm^2^ (**d**) and percent nuclear area covered by aggregates (**e**) in mock- and α-amanitin-injected embryos at 5.0 hpf. Particles/μm^2^ and percent nuclear area were measured in nuclei from each of four α-amanitin- and four mock-injected embryos. Each graphed data point represents data from one embryo; values for each embryo are the average of six to ten representative nuclei. Error bars indicate SD. Source data for panel a is provided as a source data file

We noted that the timing of H3K9me3 establishment and chromatin compaction in the zebrafish embryo roughly coincided with the MZT, which occurs during the tenth cell division in zebrafish (3 hpf). This critical transition in embryonic development marks the stage at which the zygotic genome is activated for the first time, and maternally deposited RNAs are degraded. To test whether blocking zygotic transcription would impact heterochromatin establishment during zebrafish embryogenesis, we injected embryos with the RNA polymerase II inhibitor α-amanatin at the one-cell stage and assessed H3K9me3 levels at 4.5 hpf. At this time point, α-amanatin-injected embryos exhibited strong reductions in total H3K9me3 levels and reduced H3K9me3 enrichment at pericentromeric repeats relative to controls (Fig. 3a, b, Supplementary Fig. 3a). Similar results were obtained using triptolide, an unrelated inhibitor of RNA polymerase II-dependent transcription (Supplementary Fig. 3b, c). In the absence of zygotic transcription, electron-dense aggregates indicative of condensed chromatin ultrastructure were also essentially undetectable in TEM images of nuclei from embryos at 5 hpf, whereas these aggregates were readily detected in mock-injected controls (Fig. 3c–e). These findings demonstrate that blocking zygotic transcription leads to impaired H3K9me3 establishment and chromatin compaction during early embryogenesis. This effect cannot be explained by the regulation of H3K9 methyltransferase transcript levels, as RNAs encoding known H3K9 methyltransferase enzymes are present at comparable or slightly higher levels prior to zygotic genome activation (ZGA) (Supplementary Fig. 3d).

### miR-430 is required for heterochromatin establishment

**Fig. 4:**
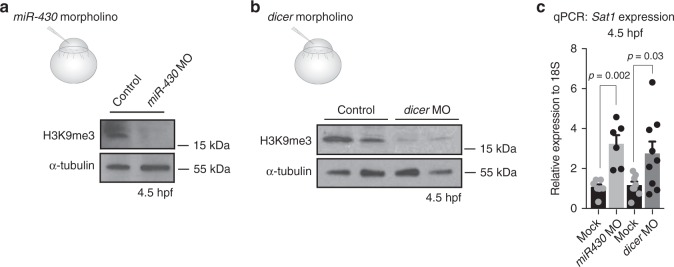
miR-430 is required for heterochromatin establishment. **a** Western blot for H3K9me3 (top) and α-tubulin (bottom) in embryos that were either mock injected or injected with 2 ng of miR-430 morpholino at the one-cell stage. Protein was collected for analysis at 4.5 h post fertilization (hpf). **b** Western blot for H3K9me3 (top) and α-tubulin (bottom) using protein extracted from embryos that were either injected with 2 ng of control morpholino or injected with dicer morpholino at the one-cell stage. Protein was collected for analysis at 4.5 hpf. **c** Satellite-1 (Sat1) pericentromeric transcript levels in 4.5 hpf embryos that were either injected with control morpholino or injected with miR-430 or dicer morpholino at the one-cell stage. *P *values were calculated using the Student’s *t* test; error bars indicate SEM. Source data for panels a and b is provided as source data files

Next, we investigated whether degradation of maternal RNA was required for heterochromatin establishment during MZT. The microRNA miR-430 is required for the degradation of maternal transcripts during zebrafish MZT, and transcription of miR-430 is dependent on ZGA^14^. Antisense morpholino targeting miR-430 was injected into embryos at the one-cell stage, and the impact on H3K9me3 establishment was then assessed. At 5 hpf, embryos injected with miR-430 morpholino exhibited sustained expression of genes that are normally downregulated at MZT, suggesting the effectiveness of the morpholino (Supplementary Fig. 4a). Injected embryos also exhibited lower global levels of H3K9me3 at 4.5 hpf when compared to siblings, as well as a derepression of transcripts from pericentromeric repeats (Fig. 4a, c, Supplementary Fig. 4b). Similar results were obtained when we depleted the exoribonuclease Dicer, which is required for miR-430 processing (Fig. 4b, c, Supplementary Fig. 4c). These findings suggest that miR-430 is important for the establishment of H3K9me3 at MZT and implicate the degradation of maternal mRNA in the developmental regulation of heterochromatin establishment.

### miR-430 mediates clearance of maternal Smarca2 RNA

**Fig. 5:**
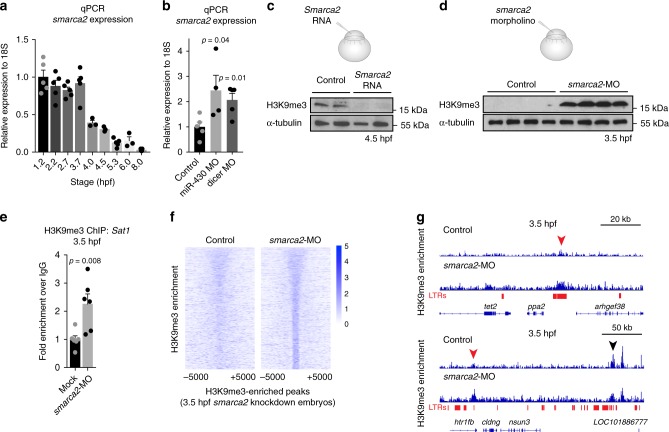
Smarca2 degradation is required for H3K9me3 establishment. **a** Quantitative reverse transcription-PCR (RT-PCR) showing *smarca2* RNA decreases during maternal to zygotic transition (MZT). Error bars indicate SEM. **b** Quantitative RT-PCR confirming elevated levels of *smarca2* transcripts in 5 h post fertilization (hpf) embryos that were injected with 2 ng of miR-430 or dicer morpholino at the one-cell stage. *P* values were calculated using Student’s *t* test; error bars indicate SEM. **c** Western blot for H3K9me3 (top) and α-tubulin (bottom) in embryos that were either injected with 200 pg of in vitro transcribed antisense RNA or *Smarca2* mRNA at the one-cell stage. Protein was collected for analysis at 4.5 hpf. **d** Western blot for H3K9me3 (top) and α-tubulin (bottom) in embryos that were either mock injected or injected with 2 ng of *smarca2* morpholino at the one-cell stage. Protein was collected for analysis at 3.5 hpf. **e** Chromatin immunoprecipitation with sequencing (ChIP) for H3K9me3 enrichment at Satellite-1 (Sat1) sequences in embryos injected with a green fluorescent protein (GFP) control morpholino or with *smarca2* morpholino. Analysis was performed at 3.5  hpf. *P* values were calculated using the Student’s *t* test; error bars indicate SEM. **f**–**g**) ChIP for H3K9me3-enrichment in embryos injected with a scrambled control morpholino or morpholinos targeting *smarca2*. **f** Heatmap depicting H3K9me3 enrichment in control embryos (left) across all H3K9me3 peak centers (±5000  bp) identified in *smarca2* morpholino-injected embryos (right). **g** Screen shots of two representative genomic regions. Red arrows indicate regions that gain H3K9me3 in *smarca2* morpholino-injected samples. The black arrow indicates a region that is enriched for H3K9me3 in both control and morpholino-injected embryos. Source data for panels c and d is provided as a source data files

To identify potentially relevant miR-430 targets, we turned to previous work by Giraldez et al.^14^, which identified the subset of maternal transcripts that are degraded by miR-430. Interrogation of this dataset revealed that *smarca2*, an ATP-dependent chromatin remodeler, was among the five genes that showed the most significant elevation in transcript levels after miR-430 processing was blocked. We confirmed that *smarca2* 3′-untranslated region contained multiple miR-430 binding sites (Supplementary Fig. 4d). Consistent with these findings, we found that *smarca2* transcripts were downregulated in wild-type embryos during MZT, and that following MZT, embryos injected with miR-430 or dicer morpholino had elevated levels of *smarca2* compared to mock-injected controls (Fig. 5a, b). Downregulation of Smarca2 at MZT is also observed in published RNA-Seq data (Supplementary Fig. 5a)^15^.

### Smarca2 RNA degradation is required for H3K9me3

To determine whether prolonged expression of *smarca2* in the developing embryo would impact heterochromatin establishment, we injected one-cell stage embryos with *Smarca2* mRNA lacking miR-430 recognition sites, and assayed H3K9me3 levels at the dome stage (4.5 hpf). Consistent with a role for Smarca2 in inhibiting heterochromatin establishment, we found that, at the dome stage, embryos that had been injected with miR-430-resistant RNA encoding *Smarca2* had reduced H3K9me3 levels compared to mock-injected controls (Fig. 5c, Supplementary Fig. 5b). Conversely, knockdown of *smarca2* using two independently designed morpholinos was sufficient to accelerate the onset of H3K9me3 incorporation (Fig. 5d, Supplementary Fig. 5c, d). We observed strong H3K9me3 signal by western blot in morpholino-injected embryos at 3.5 hpf, while H3K9me3 signal was not observed in wild-type embryos at this stage. Precocious H3K9me3 incorporation was associated with abnormal development at 24 hpf, using either of the two smarca2 morpholinos (Supplementary Fig. 6a, c). All abnormal embryos were dead by 7 dpf.

Precocious accumulation of H3K9me3 was noted at pericentromeric Sat1 repeats by ChIP, suggesting that Smarca2 depletion allowed for early incorporation of H3K9me3 at sequences that were destined for later enrichment in wild-type embryos (Fig. 5e). Consistent with this model, genome-wide ChIP-seq analysis of *smarca2* morpholino-injected embryos at 3.5 hpf identified 4692 H3K9me3 peaks that were not present in control embryos, with roughly 90% of these precocious peaks overlapping annotated repeats (Fig. 5f, g, Supplementary Fig. 7a). ChIP-seq profiles from embryos injected with Smarca2 clustered with profiles from 4.5 and 6 hpf embryos, whereas controls were clustered with profiles from 2.5 hpf embryos (Supplementary Fig. 7b).

**Fig. 6:**
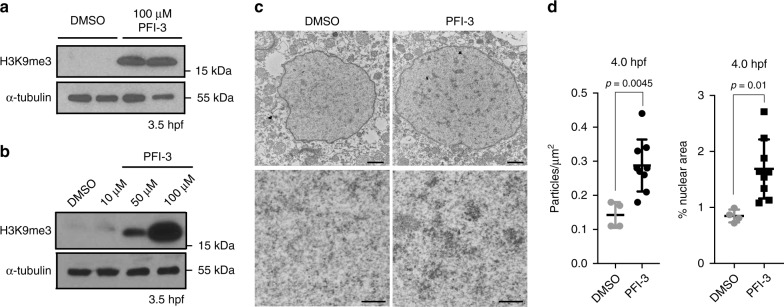
Smarca2 inhibition accelerates the timeline of chromatin compaction. **a** Western blot for H3K9me3 (top), and α-tubulin (bottom) in embryos that were either mock injected or injected with 100 μM of the Smarca2 inhibitor PFI-3 at the one-cell stage. Protein was collected for analysis at 3.5 h post fertilization (hpf). **b** Western blot using embryos injected with increasing concentrations of PFI-3. Protein was collected for analysis at 3.5 hpf. **c** Electron micrographs demonstrating increased levels of chromatin compaction in 4.5 hpf embryos that were injected with dimethyl sulfoxide (DMSO) or 100 μM PFI-3 at the one-cell stage. Bottom panels represent higher magnification images (×20,000) of nuclear interior in mock- and PFI-3-injected embryos at specified time points. All scale bars indicate 1 μm. **d** Quantification of the number of particles per nuclear μm^2^ and percent nuclear area. Each data point indicates an individual embryo, for each embryo (four DMSO and nine PFI-3-injected embryos) values for particles per um^2^ and percent nuclear area were averaged from five to ten representitive nuclei. *P* values were calculated using the Student’s *t* test; error bars indicate SD. Source data for panels a and b is provided as a source data files

To confirm these results using a morpholino-independent approach, we inhibited Smarca2 function in the early embryo using the Bromo domain binding small-molecule PFI-3, which can displace the Smarca2 Bromo domain from chromatin^16^. We found that PFI-3 injection led to precocious establishment of H3K9me3 and abnormal development similar to that observed in morpholino experiments (Fig. 6a, Supplementary Fig. 6a–c). The effect on H3K9me3 was dose dependent (Fig. 6b and Supplementary Fig. 5e)

### Smarca2 inhibition accelerates condensed chromatin formation

**Fig. 7:**
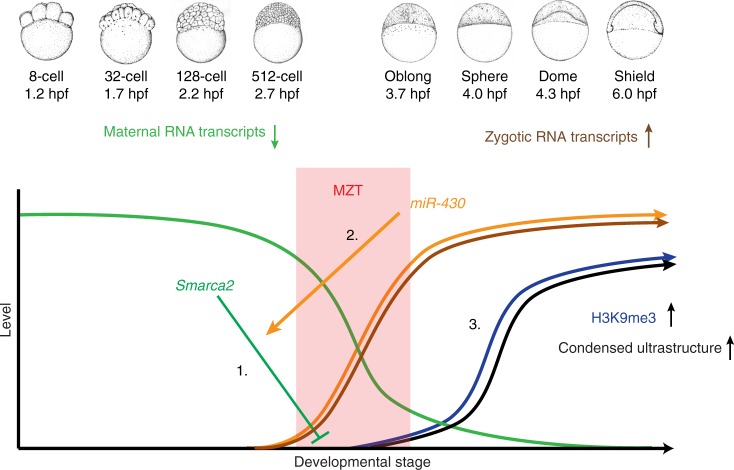
Model for Smarca2-miR430-dependent heterochromatin formation. The early embryo relies exclusively on maternally deposited RNA transcripts and protein, and only starts transcribing the zygotic genome at the maternal to zygotic transition. This model shows that maternal *smarca2*, a catalytic subunit of the BRG1/BRM-associated factor (BAF) complex, inhibits H3K9me3 incorporation and chromatin condensation prior to the maternal zygotic transition (1). At the onset of zygotic transcription, the microRNA miR-430 is transcribed and targets maternal *smarca2* for degradation (2). Decreasing levels of *smarca2* are sufficient to allow for the incorporation of H3K9me3 and formation of condensed chromatin ultrastructure post maternal to zygotic transition (MZT) (3). Zebrafish embryonic stage schematics are reproduced from Kimmel et al.^22^ with permission from John Wiley & Sons Inc

Finally, we asked whether early establishment of H3K9me3 following inhibition of Smarca2 would be sufficient to promote the precocious establishment of condensed chromatin ultrastructure in the developing embryo. To this end, embryos were injected with PFI-3 at the one-cell stage and embryonic nuclei were examined by TEM at 4 hpf. At this stage, we found that embryos that had been injected with PFI-3 had significantly more heterochromatic aggregates than mock-injected controls, and that these aggregates covered a larger percent of nuclear area in PFI-3-injected embryos compared to controls (Fig. 6c, d). These observations combined with the molecular studies described above suggest that Smarca2 serves as a maternally supplied inhibitor of heterochromatin establishment during early embryogenesis. Collectively, our findings identify MZT and the miR-430/Smarca2 axis as an important mechanism controlling the de novo establishment of vertebrate heterochromatin in the early embryo (Fig. 7).

## Discussion

Reprogramming of H3K9me3 marked constitutive heterochromatin has been widely noted in metazoa. However, very little is known regarding the mechanisms that govern this process, especially in vertebrate systems^5–7^. In this study, we demonstrate that global establishment of H3K9me3 marked heterochromatin is controlled by the MZT in the zebrafish embryo and we identify Smarca2 as an essential gatekeeper of H3K9me3 establishment and global chromatin compaction during zebrafish embryogenesis. In addition to identifying MZT as a key regulator of heterochromatin establishment, these studies identify maternal inhibition, rather than targeting of the de novo methyltransferase machinery as a primary mechanism controlling de novo establishment of H3K9me3 in the embryo. Our results also demonstrate that precocious establishment of H3K9me3 is sufficient to accelerate the timeline of chromatin compaction at the ultrastructure level, implicating H3K9me3 deposition as a primary barrier to heterochromatin establishment in the early embryo.

The extent and timing of heterochromatin reprogramming varies significantly between species. For example, heterochromatin depletion in the early mouse embryo preferentially affects the paternal genome, with major gains in H3K9me3 noted by the two-cell stage^4,5^. In contrast, immunohistochemistry suggests that the early *C. elegans* embryo is broadly depleted for H3K9me3, with significant gains in heterochromatic markers noted in 5–20-cell stage embryos^7^. Our studies reveal that the zebrafish embryo is globally deficient in H3K9me3 and condensed chromatin ultrastructure until at least the 1000-cell stage. This observation indicates that zebrafish undergo roughly 10 rounds of embryonic cell division in the absence of condensed chromatin ultrastructure. This extensive and prolonged heterochromatin depletion raises questions regarding how genome integrity is sustained during these early rounds of rapid cell division.

The coupling of heterochromatin establishment and the maternal zygotic transition in zebrafish assures a globally decondensed genome at the onset of ZGA. This open structure has the potential to facilitate initiation of ZGA and to reduce global barriers to transcription factor binding during the critical window immediately following ZGA. It is possible that the benefits of increased chromatin plasticity during this period outweigh the potential costs of increased genome instability associated with heterochromatin depletion. Indeed, we find that precocious accumulation of H3K9me3 is associated with abnormal development in embryos injected with Smarca2 morpholino or PFI-3. The timing of heterochromatin establishment roughly correlates with MZT in other species as well, raising the possibility that MZT may be a conserved regulator of heterochromatin establishment. Supporting this hypothesis, blocking zygotic transcription disrupts replication timing in the Drosophila embryo and spatial reorganization of pericentromeric domains in the mouse embryo, two features that are associated with heterochromatin^17,18^.

In this study, we identify zygotic transcription of miR-430 and subsequent clearance of *Smarca2* mRNA as a key mechanism tying MZT to heterochromatin establishment. There have been few previous reports identifying potential mechanisms regulating the establishment of H3K9me3 marked heterochromatin during development in any species. In mouse, a single small interfering RNA-based study has implicated the chromatin assembly factor 1a in the regulation of H3K9me3 establishment and silencing at a subset of LTR transposons^5^. However, global heterochromatin formation was unaffected in these embryos. Mutation of Lsd1/Su(VAR)3-3 was shown to reduce H3K9me3 establishment at pericentromeric sequences in Drosophila, but similar effects were not reported in mice^6,19^. More recently, nuclear accumulation of the histone methyltransferase Setdb1/MET-2 was implicated in regulating the timing of heterochromatin establishment in *C. elegans*^7^. While it remains to be seen whether this pathway is also conserved in vertebrate systems, there is potential for the miR-430/Smarca2 regulatory axis to act upstream of Met-2 and other as yet unidentified targeting pathways to tightly control the precise window of heterochromatin establishment.

Smarca2 and the related Smarca4 protein function as alternative catalytic subunits for the BRG1/BRM-associated factor (BAF) complex, which is broadly involved in nucleosome remodeling. BAF complexes containing Smarca4 as the catalytic subunit have been shown to inhibit establishment of H3K27me3, a known marker of facultative heterochromatin^20^. To our knowledge, Smarca2 has not been previously implicated in vertebrate heterochromatin regulation. Our findings raise the possibility that BAF may have broad functions in antagonizing the establishment of repressive chromatin states, with complex specificity designated by subunit composition. Although Smarca2 is downregulated at MZT, it is expressed at low levels in many adult tissues^21^, raising the possibility that Smarca2 may also antagonize H3K9me3 in other somatic contexts or disease states.

## Methods

### Maintenance and staging of zebrafish embryos

Zebrafish husbandry and care were conducted in full accordance with animal care and use guidelines with ethical approval by the Institutional Animal Care and Use Committees at Memorial Sloan Kettering Cancer Center and the University of Georgia. Zebrafish were raised and maintained under standard conditions in compliance with relevant protocols and ethical regulations. Fertilized eggs were obtained from the zebrafish AB strain by breeding single pairs. Embryos were reared in system water at 28.5 °C and staged according to morphology^22,23^.

### Microinjection

For all microinjection experiments, embryos were collected within 5 min of egg laying.

### Morpholino injection

Antisense morpholino oligonucleotides were purchased from Genetools. Lyophilized morpholino was diluted to a 1 mM stock solution in ddH_2_O. For injection, a fresh 1:4 dilution of stock in ddH_2_O/phenol red was prepared and heated at 70 °C for 5 min. One to two nanoliters of this solution was injected into one-cell stage embryos. Morpholino sequences are included in Supplementary Table 1.

### *Smarca2* mRNA injection

A plasmid containing the mouse *Smarca2* complementary DNA (cDNA) clone was purchased from Transomic Technologies (clone ID BC075641, IMAGE ID 30544092). The plasmid was linearized with *Sca*I and transcribed with T7 polymerase (sense RNA) or T3 polymerase (antisense RNA control) using the mMessage Machine Kit (Thermo Fisher) according to the manufacturer’s instructions (Invitrogen/Thermo Fisher). RNA was purified using G-50 columns (GE Healthcare) and 200 pg *smarca2* RNA or control antisense RNA was injected into embryos at the one-cell stage.

### Drug treatments

α-Amanitin was purchased from Sigma (A2263-1 mg) and dissolved in 1 ml ddH_2_O. For injection, a 1:20 solution was freshly prepared in ddH_2_O, and 2 nl (0.2 ng) was injected into embryos at one-cell stage. Triptolide (PG490- 1 mg, Selleckchem) was diluted in dimethyl sulfoxide (DMSO) to obtain a 2.5 mM stock. For drug treatment, the stock was further diluted to 10 mM in embryo medium. Manually dechorionated embryos were reared in the triptolide solution from the 128-cell stage until 4.3 hpf. PFI-3 (catalog no. S7315-5 mg, Selleckchem) was dissolved in DMSO to a 5 mM stock solution. For drug treatment, dilutions of PFI-3 stock in ddH_2_O (1–100 mM) were freshly prepared, thoroughly vortexed, and 1–2 nl was injected into one-cell stage embryos.

### Western blot

For protein samples, 10–20 embryos were dechorionated manually and allowed to develop to the desired stage. At the appropriate time point, embryos were manually deyolked and snap frozen in liquid nitrogen. Cells were lysed in RIPA buffer containing HALT protease inhibitors for 20 min on ice and cell lysates were then centrifuged for 20 min at 20,000 × *g*. Supernatant was used for western blot analysis. Samples were run on 12, 18, or 4–20% acrylamide gels. For all blots, α-tubulin was used on the same membrane as a loading control. All western blots are representative of at least three biological replicates. Antibodies used include: anti-H3K9me3 antibody (Abcam ab8898, 1:1000), anti-H3 antibody (Abcam, ab1791, 1:5000), anti-α-tubulin (Sigma T6074, 1:2000). H3 histone bands were confirmed to run at 17 kDa and a tubulin to run at 55 kDa using PageRuler prestained ladder (Thermo Fisher). Western blots in the figures have been cropped to include only the relevant region, full uncropped blots are available as a Source Data file.

### Chromatin immunoprecipitation

One hundred and fifty to two hundred embryos were used for preparing chromatin. Chromatin was subsequently incubated with anti-H3K9me3 antibody (Abcam ab8898, 2 μg) or an immunoglobulin G control (Abcam ab15008, 2 μg) and pulled down using DYNA beads (Thermo Fisher). DNA was purified using QIAquick PCR Purification Kit and amplified using ChIP primers included in Supplementary Table 2.

ChIP-seq Libraries were constructed by end repair and A-tailing using the NEB End Repair Module (cat. # E7546S). Illumina adaptors were ligated to repaired DNA molecules using T4 ligase (NEB; cat. # M0202S). Ligation products were amplified to generate dual-indexed libraries using NEBNext Ultra II Q5 Hot Start HiFi PCR Master Mix (cat. # M0543S). Libraries were pooled and sequenced on a NextSeq500 instrument at the Georgia Genomics Facility to generate single-end 75 bp reads. Short reads (<20 bp) and adaptor sequences were removed using TrimGalore (version 0.4.4)^24^, cutadapt version 1.14^25^, and Python 2.7.8, with fastqc command (version 0.11.3). Trimmed Illumina reads were aligned to the current zebrafish genome assembly (accession # GCF_000002035.6) using BWA (version 0.7.15)^26^, with algorithms aln, and samse, which randomly assign multi-mapped reads to a single location. Duplicate reads were marked with Picard tools (version 2.4.1) (http://broadinstitute.github.io/picard) and duplicate reads were removed using samtools^27^. To plot the relative distribution of mapped reads, read counts were determined for each 25 bp window across the genome using igvtools and data were displayed using the Integrated Genome Viewer^28^. The Hypergeometric Optimization of Motif EnRichment (HOMER) software package (version 4.8)^29^ was used to identify H3K9me3 peaks in 6.5 hpf embryos and *smarca2* knockdown embryos using “findPeaks.pl” with the following parameters: -style histone -size 1000. Bedtools “intersect” (version 2.26.0)^30^ was used compare peak locations between samples and to determine the fraction of peaks that intersected with annotated repeats. HOMER was also used to construct metaplots or heatmaps centered on the H3K9me3 peaks identified in the combined 6.0 hpf sequence data using “annotatePeaks.pl” with the following options: -hist 50 -size 10,000 and with or without the -ghist flag to make heatmaps and metaplots, respectively. Heatmaps were constructed with R using the pheatmap package. The 95th percentile value was set as the maximum value and pheatmap was used to generate row-normalized heatmaps using HOMER -ghist matrix files as input. Hierachial clustering was achieved using DiffBind^31^. Raw sequence data associated with Figs. 1e, f and 4f, g are available through the NCBI GEO database (accession # GSE113086).

### RNA extraction from embryos, cDNA synthesis, and qPCR

Ten to thirty embryos were collected and total RNA was extracted using TRIzol Reagent (15596018, Ambion by Life Technology). For *Sat1* expression, RNA was subsequently treated with DNAse using the Turbo DNAse-free^TM^ (AM1907, invitrogen by Thermo Fisher Scientific). cDNA was synthesized from total RNA using GoScript™ Reverse Transcription System (REFA5000, Promega). Finally, quantitative real-time PCR was performed using Power SYBR Green PCR Master Mix (REF 4367659, Thermo Fisher) and the Thermal Cycler System. Specific amplification of the PCR products was confirmed after analyzing the dissociation curve. 18S primers were chosen for normalization based on McCurley and Callard^32^. Primers are included in Supplementary Table 2.

### Immunohistochemistry

Embryos were manually dechorionated and fixed in 4% paraformaldehyde/0.1% phosphate-buffered saline (PBS)-Tween20 (PBST) at 4 °C overnight. The samples were washed with PBST three times for 10 min, and incubated in blocking solution (10% goat serum, 1% DMSO, 0.1% Tween-20 in PBS) for 2 h at room temperature. This step was followed by incubation of primary antibody in block (Abcam ab8898, 1:100–1:200) overnight at 4 °C. Subsequently, embryos were washed with PBST three times for 15 min and then incubated with Alexa 546-conjugated anti-rabbit secondary antibody (1:1000, Invitrogen/Thermo Fisher) for 2 h at room temperature. After washing the embryos three times for 10 min in PBST, staining was analyzed immediately using a SP5 confocal microscope (Leica).

### Electron microscopy

Dechorionated embryos were fixed at 4 °C for one day in modified Karmovskys fix of 2.5% glutaraldehyde, 4% parafomaldehye, and 0.02% picric acid in 0.1 M sodium caocdylate buffer at pH 7.2. Following a secondary fixation in 1% osmium tetroxide and 1.5% potassium ferricyanide, samples were dehydrated through a graded ethanol series, and embedded in an epon analog resin. Ultrathin sections were cut using a Diatome diamond knife (Diatome, USA, Hatfield, PA, USA) on a Leica Ultractu S ultramicrotome (Leica, Vienna, Austria). Sections were collected on copper grids and further contrasted with lead citrate and viewed on a JEM 1400 electron microscope (JEOL, USA Inc., Peabody, MA, USA) operated at 100 kV. Images were recorded with a Veleta 2 K × 2 K digital camera (Olympus-SIS, Germany). Image analysis was performed using ImageJ with the Illumination bias plugin (http://sites.imagej.net/Julien.pontabry/plugins/)^10^. Nuclei were manually selected and electron-dense and non-electron-dense regions within nuclei were identified using maximum entropy thresholding tools. Following thresholding, particles with a minimum size of 0.03 μm^2^ were automatically selected and counted. For each image, the number of particles and their total area was then expressed relative to total nuclear area.

### Statistical analysis

All statistical analysis was carried out using GraphPad Prism version 7.000 (GraphPad Software, La Jolla, CA, USA). All tests were two-sided, unless otherwise indicated.

### Reporting summary

Further information on experimental design is available in the Nature Research Reporting Summary linked to this article.

## Supplementary information

Supplementary Information

Peer Review File

Reporting Summary

Source Data

## Data Availability

A reporting summary for this Article is available as a Supplementary Information file. The ChIP-seq data sets generated and analyzed during the current study are available in NCBI GEO (accession # GSE113086). All other datasets generated during and/or analyzed during the current study are available from the corresponding author on request. The source data underlying images of all western blots are provided as a Source Data file.
