# Supplementary material for: The maternal to zygotic transition regulates genome-wide heterochromatin establishment in the zebrafish embryo
Source: Nat Commun. 2019 Apr 4;10:1551. doi: 10.1038/s41467-019-09582-3 (PMC6449393; doi:10.1038/s41467-019-09582-3)
Supplement: Supplementary file 1 — Supplementary Information [file 41467_2019_9582_MOESM1_ESM.pdf]

The maternal to zygotic transition regulates genome-wide heterochromatin establishment in the zebrafish embryo

Laue et al.

Supplemental Information

Supplementary Figures

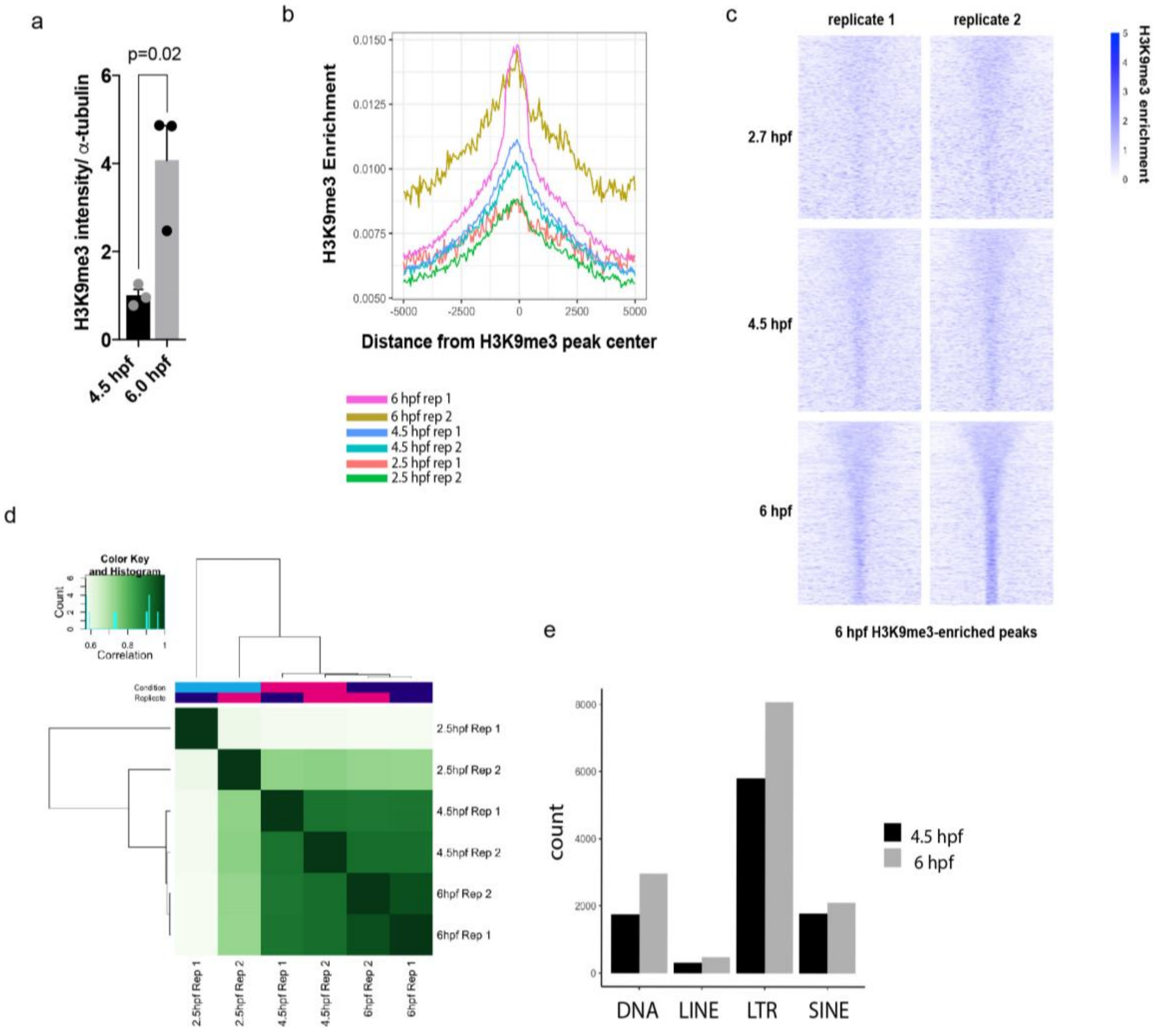

Supplementary Figure 1. Early stage zebrafish embryos are depleted for H3K9me3

(a) Ratio of H3K9me3 levels relative to α-tubulin at 4.5 and 6.0 hpf as measured by western blot. Band intensity was measured for H3K9me3 and α-tubulin on the same blot using image J software, and ratios were calculated for individual samples. Bars reflect average ratios from

three samples. Error bars indicate standard error of the mean (SEM) (b) Plot showing the normalized average enrichment profile of H3K9me3 at 17,621 peaks identified in 6 hpf embryos. The HOMER software package was used to create the metaplot using a window size of 50 base pairs across a 10 kilobase region centered on each enriched peak. Differences between 6 hpf samples may reflect variation in ChIP efficiency or minor differences in developmental staging between the two 6 hpf replicates. (c) Heat maps depict row-normalized H3K9me3 enrichment for two replicate samples from each developmental time point (2.7 hpf, 4.5 hpf and 6 hpf) across the same 17,621 H3K9me3-enriched peaks shown in a. Peaks are ordered from largest (top) to smallest (bottom). (d) Clustering by Chip-dif demonstrates samples from the same time point are most related to each other. (e) Bar graph depicting the number of H3K9me3 peaks detected in different transposon classes at 4.5 and 6 hpf.

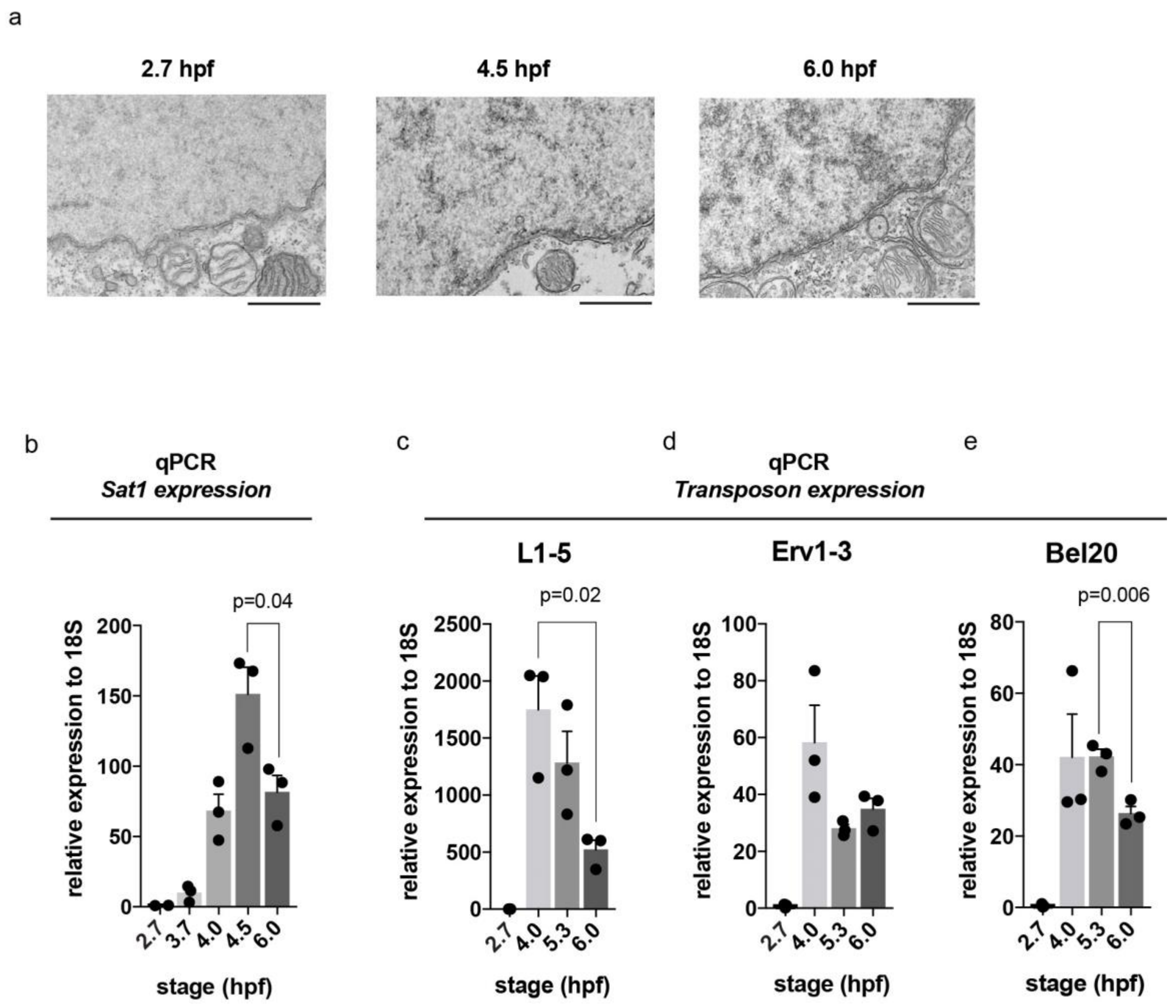

Supplementary Figure 2. Elevated repetitive transcripts at stages with less condensed chromatin ultrastructure (a) TEM images showing nuclear periphery and cytoplasmic organelles at 2.7, 4.5 and 6.0 hpf. Scale bar equals 1 μm. (b-e) Quantitative RT-PCR showing increasing levels of transcripts derived from repetitive elements following the onset of zygotic transcription. A decline in transcript levels at 6.0 hpf correlates with the onset of heterochromatin formation. All p-values were calculated using the students t-test, error bars indicate SEM.

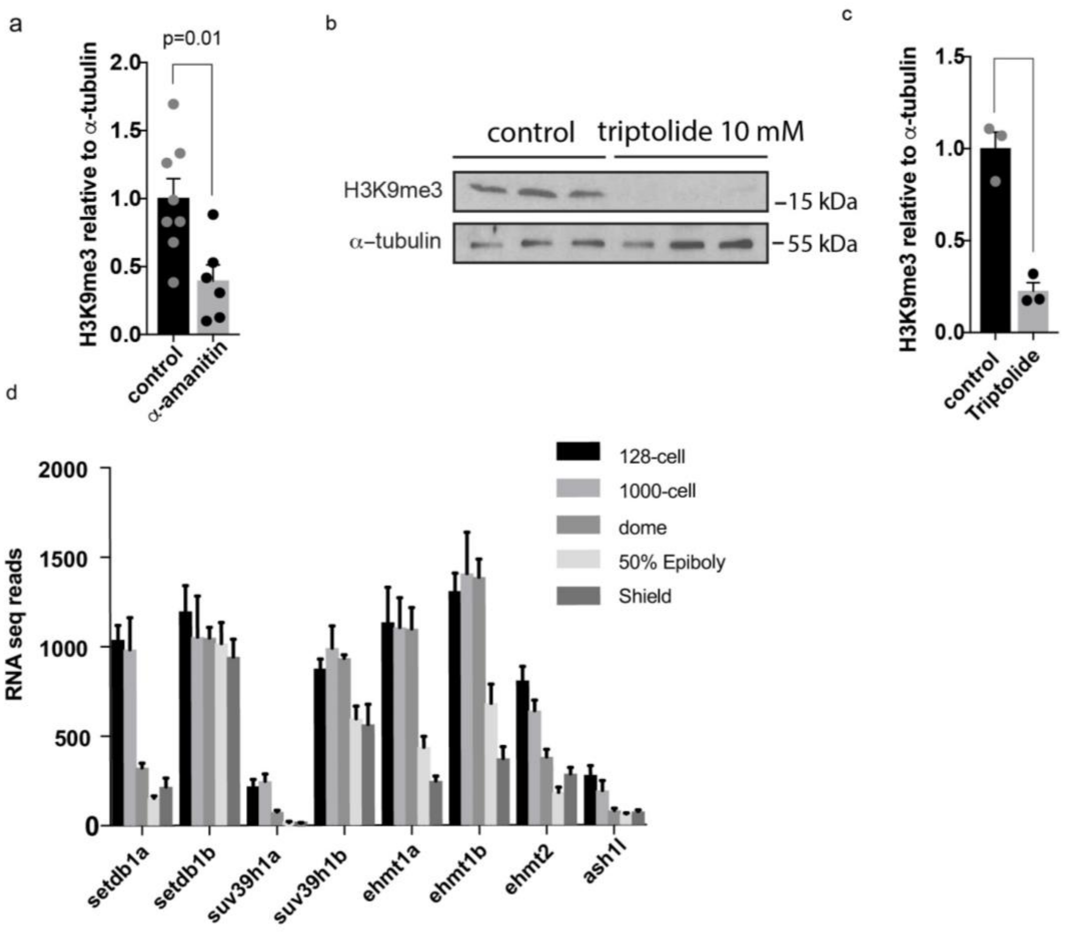

Supplementary Figure 3. Blocking zygotic transcription is associated with reduced H3K9me3 (a) Ratio of H3K9me3 to α-tubulin for western blots using protein extracted from embryos that were either mock-injected or injected with 0.2 ng of α -amanitin at the 1-cell stage. Protein was collected at 4.5 hpf. p-values was calculated using the student's t test. Variability in the extent of H3K9me3 depletion is likely explained by variable effectiveness in inhibition of ZGA. (b) Western blot analysis for H3K9me3 and α -tubulin using protein extracted from embryos that were either reared in embryo medium or medium containing 10mM triptolide starting at the 128-cell stage. Protein was collected at 4.3 hpf. (c) H3K9me3 levels relative to α -tubulin for triptolide experiment at 4.3 hpf. (d) Expression profile of known H3K9 methyltransferases using published RNA-seq data [1] at time points before (128-cell) and after the maternal to zygotic transition (dome-shield stages). All error bars indicate SEM.

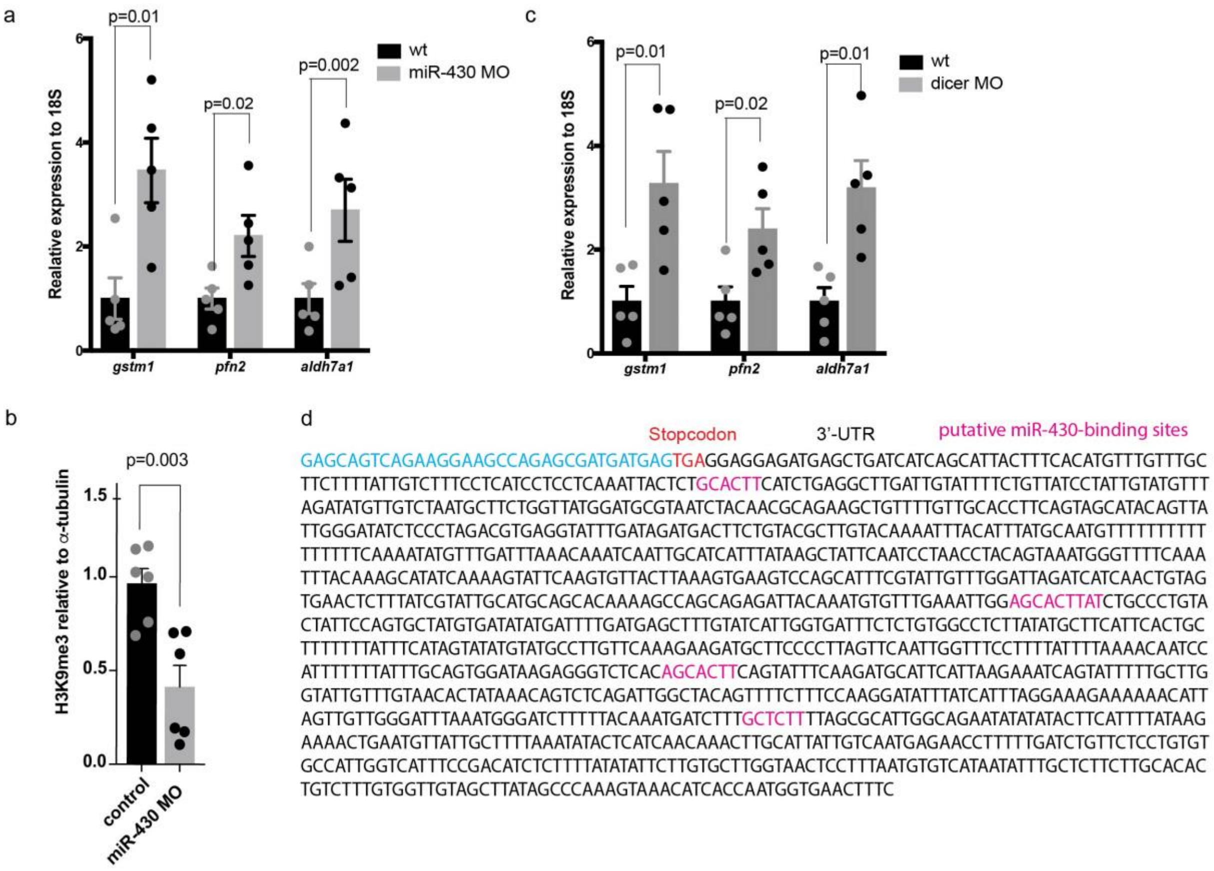

Supplementary Figure 4. Morpholino depletion of mir-430 or dicer increases maternally deposited transcripts (a) Quantitative RT-PCR assessing expression of *gstm*, *pfn2* and *aldh7a1* at 5 hpf in wildtype embryos compared to embryos injected with miR-430 morpholino. Transcripts for all three genes are normally degraded at MZT[1] . (b) Ratio of H3K9me3 to α -tubulin on western blots using protein from control embryos or embryos that were injected with 2 ng miR-430 morpholino (MO). Protein was extracted from 4.5 hpf embryos. Data from six samples are presented. (c) Quantitative RT-PCR assessing expression of *gstm*, *pfn2* and *aldh7a1* at 5 hpf in wildtype embryos compared to embryos injected with dicer morpholino. (d) Predicted miR-430 binding sites in the 3'UTR of the zebrafish *smarca2* gene using criteria described in Giraldez et al., 2006[2]. Predicted binding sites are highlighted in pink. All error bars indicate SEM.

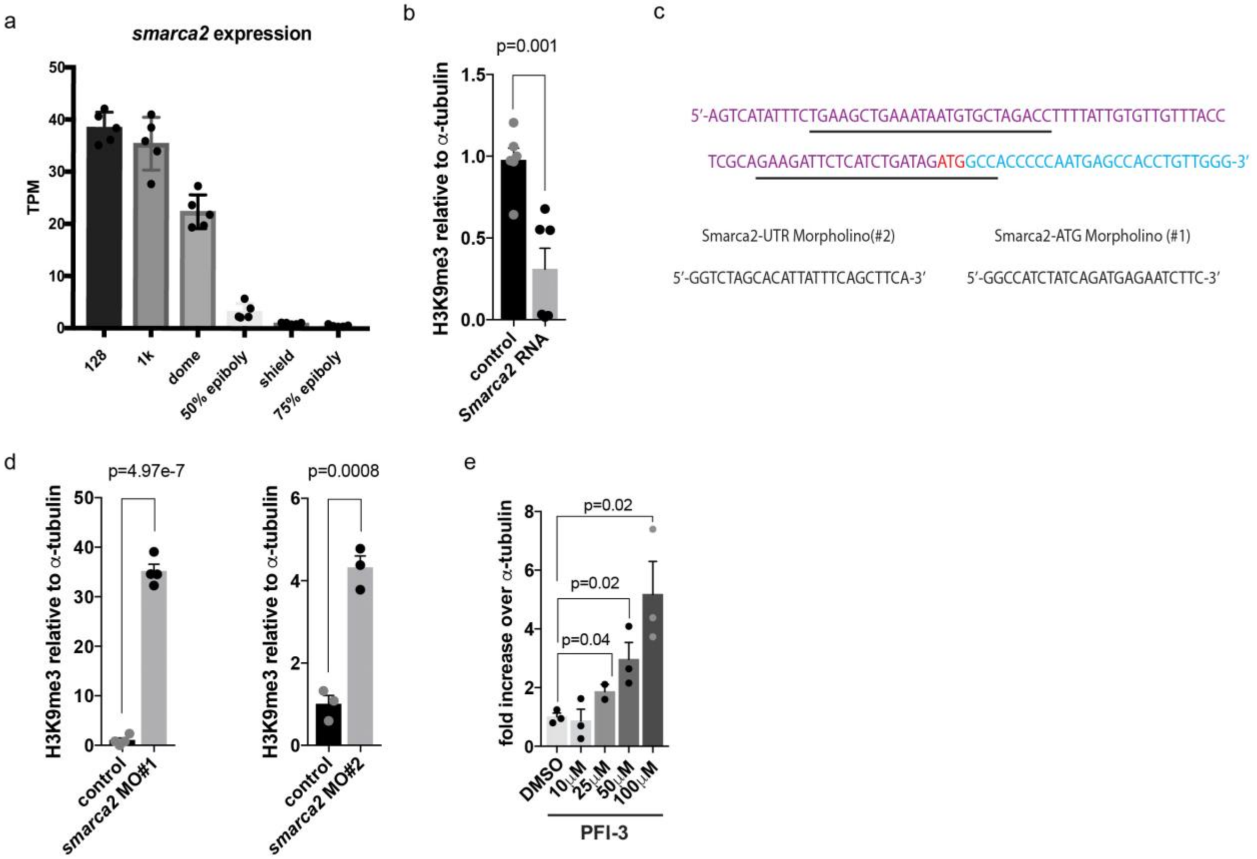

Supplementary Figure 5. Smarca2 depletion or inhibition promotes H3K9me3 establishment (a) Bar graph of Smarca2 expression based on RNA-seq data from White et al 2017 [1] (b) Ratio of H3K9me3 to α -tubulin. Embryos were injected with 200 ng control- or *Smarca2*-RNA and protein was collected at 4.5 hpf. Data from five samples is presented. (c) Schematic of the binding sites of smarca2 morpholinos in the 5' UTR and ATG region of the *smarca2* gene. (d) Ratio of H3K9me3 to α -tubulin for embryos injected with either control morpholino, Smarca2-UTR morpholino (MO #1) or Smarca2-ATG morpholino (MO #2). Protein was extracted at 3.5 hpf. (e) Dosage curve of embryos injected with increasing concentrations of PFI-3. Protein was collected for analysis at 3.5 hpf. Bars indicate the average ratio of H3K9me3 band intensity divided by α–tubulin signal intensity over three biological replicates. p values were calculated using the students t test, Error bars indicate SEM.

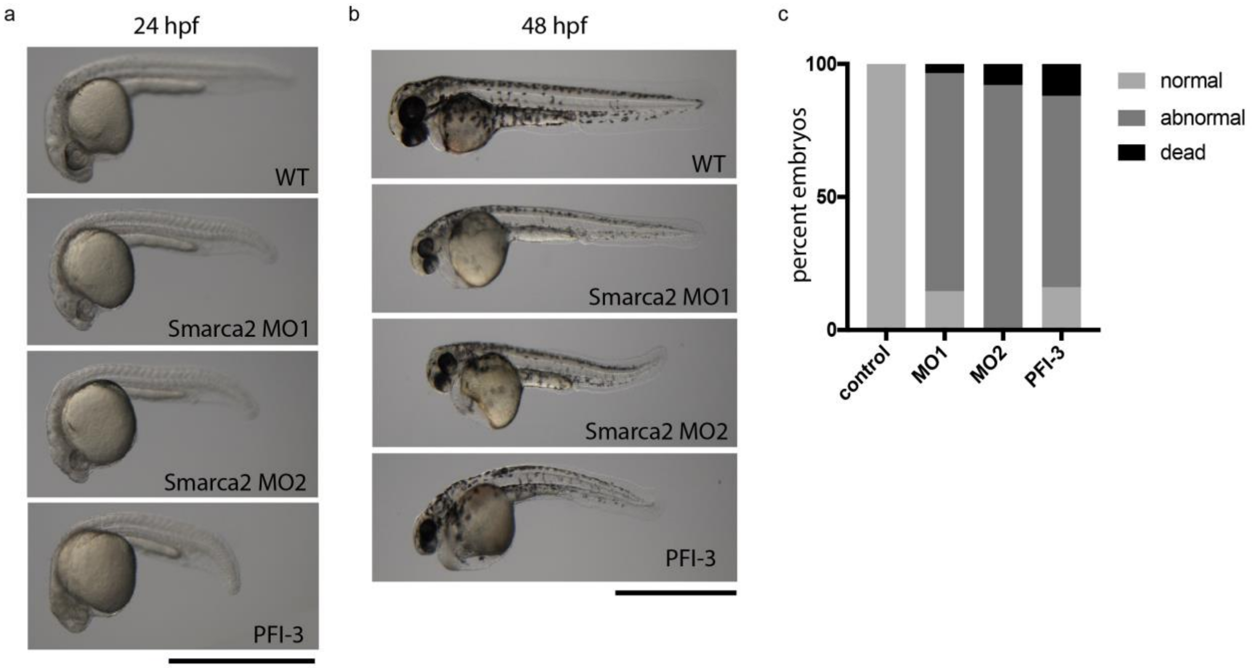

Supplementary Figure 6. Abnormal development following Smarca2 depletion or inhibition (a) Bright field images of 24 hpf zebrafish larvae following injection with 2 ng of morpholinos targeting Smarca2 (MO1 or MO2) or 100 μM PFI-3 at the 1 cell stage. Scale bars represent 1 mm. (b) Bright field images of 48 hpf zebrafish larvae following injection with 2 ng of morpholinos targeting Smarca2 (MO1 or MO2) or 100 μM PFI-3 at the 1 cell stage. Scale bars represent 1 mm. (c) Quantification of embryos that were normal, dead or showed anomalies consistent with panel a, at 24 hpf. A minimum of 70 embryos were scored per condition. Numbers are representative of three independent experiments.

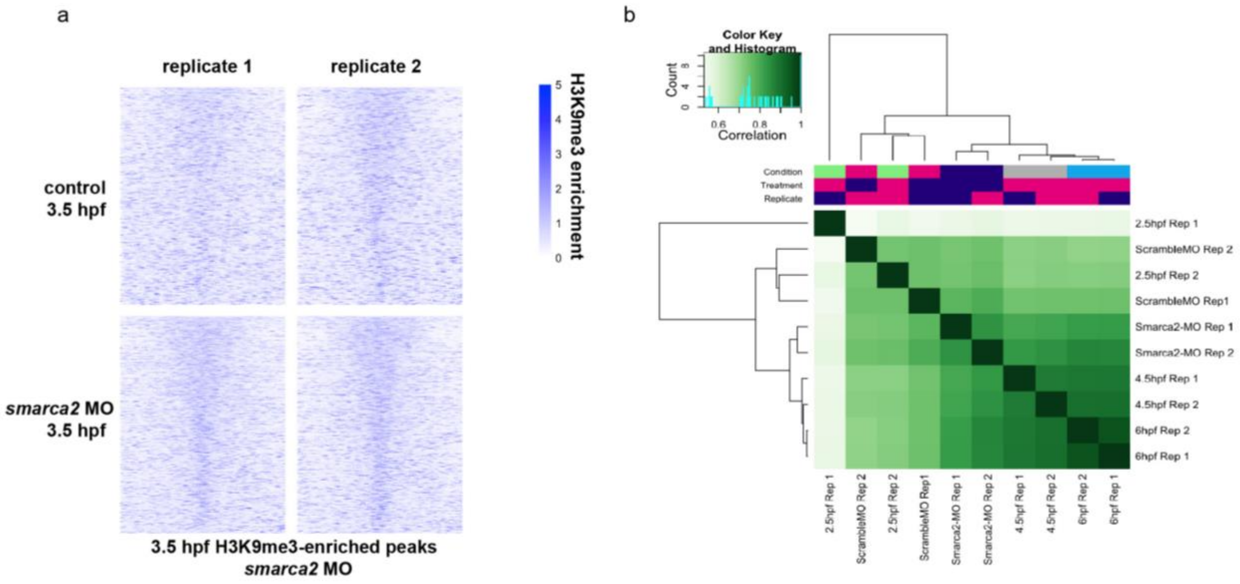

Supplementary Figure 7. Global analysis of H3K9me3 in Smarca2 morpholino depleted embryos (a) Heat maps depict row-normalized H3K9me3 enrichment for two replicate samples from embryos with control morpholino or morpholino targeting *smarca2* across H3K9me3-enriched peaks identified in the *smarca2* knockdown embryos at 3.5hpf. Peaks are ordered from largest (top) to smallest (bottom). (b) Hierarchical clustering of Chip-seq profiles from embryos injected with Smarca2 or scramble morpholino compared to wild type embryos at 2.5, 4.5 and 6.0 hpf.

**Morpholino name**	**Morpholino sequence**
control-MO	5’-ACAGCTCCTCGCCCTTGCTCTCACC-3’,
miR430-MO[3,4 5]	5’-ACTACCCCAACAAATAGCACTTACC-3’
dicer[6,7 8]	5’-CTGTAGGCCAGCCATGCTTAGAGAC-3’
*smarca2*_ATG-MO	5’-GGCCATCTATCAGATGAGAATCTTC-3’
*smarca2*_UTR-MO	5’-GGTCTAGCACATTATTTCAGCTTCA-3’

Supplementary Table 1. Morpholinos used in this study

Primer name	Primer sequence
Sat1-F	5’-TGTTTTAGACAACATTTCATGCAC-3’
Sat1-R	5’-AGTCAGCCAGCAGAGAGGTC-3’
smarca2-F	5’-CGAGTGTCCCACATACAATGC-3’
smarca2-R	5’-CATTGCTTTTTCATGGATTCC-3’
18S-F	5’-TCGCTAGTTGGCATCGTTTATG-3’
18S-R	5’-CGGAGGTTCGAAGACGATCA-3’
gstm-F	5’-GAGTTGTTGGATCAGCATCG-3’
gstm-R	5’-GCCATCTTGTTGTTCACAGG-3’
pfn2-1-F	5’-GAACATTCGCAAACATCACG-3’
pfn2-1-R	5’-CCTCCGTTGTCAGATTGTCC-3’
aldh7a1-F	5’-GAGCAGTGGAATCCTGTTGG-3’
aldh7a1R	5’-CAACACTTGTAAGGGGTGTGG-3’
ChIPSat1-F	5’-AAGCAAGTTGCAAGTGAAAATCT-3’
ChIPSat1-R	5’-AGTCAGCCAGCAGAGAGGTC-3’

Supplementary Table 2. Primers used in this study

Supplementary References

1 White, R. J. *et al.* A high-resolution mRNA expression time course of embryonic development in zebrafish. *Elife* **6**, doi:10.7554/eLife.30860 (2017).

2 Giraldez, A. J. *et al.* Zebrafish MiR-430 promotes deadenylation and clearance of maternal mRNAs. *Science* **312**, 75-79, doi:10.1126/science.1122689 (2006).

3 van Boxtel, A. L. *et al.* A Temporal Window for Signal Activation Dictates the Dimensions of a Nodal Signaling Domain. *Dev Cell* **35**, 175-185, doi:10.1016/j.devcel.2015.09.014 (2015).

4 Mishima, Y. & Tomari, Y. Codon Usage and 3' UTR Length Determine Maternal mRNA Stability in Zebrafish. *Mol Cell* **61**, 874-885, doi:10.1016/j.molcel.2016.02.027 (2016).

5 Makino, S., Mishima, Y., Inoue, K. & Inada, T. Roles of mRNA fate modulators Dhh1 and Pat1 in TNRC6-dependent gene silencing recapitulated in yeast. *J Biol Chem* **290**, 8331-8347, doi:10.1074/jbc.M114.615088 (2015).

6 Thatcher, E. J., Paydar, I., Anderson, K. K. & Patton, J. G. Regulation of zebrafish fin regeneration by microRNAs. *Proc Natl Acad Sci U S A* **105**, 18384-18389, doi:10.1073/pnas.0803713105 (2008).

7 Francia, S. *et al.* Site-specific DICER and DROSHA RNA products control the DNA-damage response. *Nature* **488**, 231-235, doi:10.1038/nature11179 (2012).

8 Wienholds, E., Koudijs, M. J., van Eeden, F. J., Cuppen, E. & Plasterk, R. H. The microRNA-producing enzyme Dicer1 is essential for zebrafish development. *Nat Genet* **35**, 217-218, doi:10.1038/ng1251 (2003).
