# Supplementary material for: The maternal to zygotic transition regulates genome-wide heterochromatin establishment in the zebrafish embryo
Source: Nat Commun. 2019 Apr 4;10:1551. doi: 10.1038/s41467-019-09582-3 (PMC6449393; doi:10.1038/s41467-019-09582-3)
Supplement: Supplementary file 3 — Reporting Summary [file 41467_2019_9582_MOESM3_ESM.pdf]

nature research

Corresponding author(s): Mary Goll

Reporting Summary

Nature Research wishes to improve the reproducibility of the work that we publish. This form provides structure for consistency and transparency in reporting. For further information on Nature Research policies, see Authors & Referees and the Editorial Policy Checklist.

Statistical parameters

When statistical analyses are reported, confirm that the following items are present in the relevant location (e.g. figure legend, table legend, main text, or Methods section).

n/a	Confirmed	
☐	☒	The exact sample size (n) for each experimental group/condition, given as a discrete number and unit of measurement
☐	☒	An indication of whether measurements were taken from distinct samples or whether the same sample was measured repeatedly
☐	☒	The statistical test(s) used AND whether they are one- or two-sided<br>*Only common tests should be described solely by name; describe more complex techniques in the Methods section.*
☒	☐	A description of all covariates tested
☐	☒	A description of any assumptions or corrections, such as tests of normality and adjustment for multiple comparisons
☐	☒	A full description of the statistics including central tendency (e.g. means) or other basic estimates (e.g. regression coefficient) AND variation (e.g. standard deviation) or associated estimates of uncertainty (e.g. confidence intervals)
☐	☒	For null hypothesis testing, the test statistic (e.g. F, t, r) with confidence intervals, effect sizes, degrees of freedom and P value noted<br>*Give P values as exact values whenever suitable.*
☒	☐	For Bayesian analysis, information on the choice of priors and Markov chain Monte Carlo settings
☒	☐	For hierarchical and complex designs, identification of the appropriate level for tests and full reporting of outcomes
☒	☐	Estimates of effect sizes (e.g. Cohen's d, Pearson's r), indicating how they were calculated
☐	☒	Clearly defined error bars<br>*State explicitly what error bars represent (e.g. SD, SE, CI)*

Our web collection on statistics for biologists may be useful.

Software and code

Policy information about availability of computer code

Data collection	no software was used to collect data
Data analysis	Software used to analyze ChIP-seq data is detailed in the materials and methods. TrimGalore (version 0.4.4), cutadapt (version 1.14), Python (version 2.7.8), fastqc (version 0.11.3), BWA (version 0.7.15), Picard tools (version 2.4.1), samtools (version 1.3), igvtools (version 2.3.81) , HOMER (version 4.8), bedtools (version 2.26.0). Rstudio (version 1.0.153) using R (version 3.4.1) pheatmap and ggplot2 was used to generate heatmaps and metaplots.

For manuscripts utilizing custom algorithms or software that are central to the research but not yet described in published literature, software must be made available to editors/reviewers upon request. We strongly encourage code deposition in a community repository (e.g. GitHub). See the Nature Research guidelines for submitting code & software for further information.

Data

Policy information about availability of data

All manuscripts must include a data availability statement. This statement should provide the following information, where applicable:

- Accession codes, unique identifiers, or web links for publicly available datasets
- A list of figures that have associated raw data
- A description of any restrictions on data availability

The ChIP-seq data sets generated and analyzed during the current study are available through the NCBI GEO database (accession # GSE113086)

Field-specific reporting

Please select the best fit for your research. If you are not sure, read the appropriate sections before making your selection.

☒ Life sciences ☐ Behavioural & social sciences ☐ Ecological, evolutionary & environmental sciences

For a reference copy of the document with all sections, see nature.com/authors/policies/ReportingSummary-flat.pdf

Life sciences study design

All studies must disclose on these points even when the disclosure is negative.

Sample size	Sample sizes were mostly limited by availability of embryos. Embryos for all experiments were collected within a 5 minute window. Each sample contained a minimum of 10 embryos, and 3 to 5 samples per experimental group were assessed.
Data exclusions	no data were excluded
Replication	each experiment was replicated at least 3 times except for ChiP seq which included two replicates per condition.
Randomization	Allocation of embryos was random.
Blinding	Data collection for EM studies were blinded.

Reporting for specific materials, systems and methods

Materials & experimental systems

n/a	Involved in the study
☒	☐ Unique biological materials
☐	☒ Antibodies
☒	☐ Eukaryotic cell lines
☒	☐ Palaeontology
☐	☒ Animals and other organisms
☒	☐ Human research participants

Methods

n/a	Involved in the study
☐	☒ ChIP-seq
☒	☐ Flow cytometry
☒	☐ MRI-based neuroimaging

Antibodies

Antibodies used	anti-H3K9me3antibody (abcam ab8898, 1:1000)<br>anti-H3 antibody (abcam, ab1791, 1:5000)<br>anti-alpha tubulin (Sigma T6074, 1:2000)
Validation	all antibodies were selected in accordance with this database:<br>Rothbart SB et al., An interactive database for the assessment of histone antibody specificity, Mol Cell. 2015 Aug 6; 59(3): 502–511.<br>anti-H3K9me3 antibody, abcam ab8898, was previously used in:<br>-Jacob V et al. DNA hypomethylation induces a DNA replication-associated cell cycle arrest to block hepatic outgrowth in uhrf1 mutant zebrafish embryos. Development 142:510-21 (2015). WB ; Zebrafish<br>-Monte E et al. Systems proteomics of cardiac chromatin identifies nucleolin as a regulator of growth and cellular plasticity in cardiomyocytes. Am J Physiol Heart Circ Physiol 305:H1624-38 (2013). WB ; Zebrafish .

anti-H3 antibody, abcam, ab1791, reacts with zebrafish according to the data sheet and has been described in several publications using zebrafish. examples see below.
-Iribarne M et al. Aipl1 is required for cone photoreceptor function and survival through the stability of Pde6c and Gc3 in zebrafish. Sci Rep 7:45962 (2017). WB ; Zebrafish .
-Ray MK et al. CAT7 and cat7l Long Non-coding RNAs Tune Polycomb Repressive Complex 1 Function during Human and Zebrafish Development. J Biol Chem 291:19558-72 (2016).
-Lin KY et al. Tumor Suppressor Lzap Suppresses Wnt/ß-Catenin Signaling to Promote Zebrafish Embryonic Ventral Cell Fates via the Suppression of Inhibitory Phosphorylation of Glycogen Synthase Kinase 3. J Biol Chem 290:29808-19 (2015). WB ; Zebrafish .

Antibodies for ChIP-seq have been previously validated by the ENCODE project consortium. Validation data are available online at: histoneantibodies.com

Animals and other organisms

Policy information about studies involving animals; ARRIVE guidelines recommended for reporting animal research

Laboratory animals: Danio rerio, AB strain

Wild animals: *Provide details on animals observed in or captured in the field; report species, sex and age where possible. Describe how animals were caught and transported and what happened to captive animals after the study (if killed, explain why and describe method; if released, say where and when) OR state that the study did not involve wild animals.*

Field-collected samples: *For laboratory work with field-collected samples, describe all relevant parameters such as housing, maintenance, temperature, photoperiod and end-of-experiment protocol OR state that the study did not involve samples collected from the field.*

ChIP-seq

Data deposition

- [x] Confirm that both raw and final processed data have been deposited in a public database such as GEO.
- [ ] Confirm that you have deposited or provided access to graph files (e.g. BED files) for the called peaks.

Data access links
May remain private before publication.

https://www.ncbi.nlm.nih.gov/geo/query/acc.cgi?acc=GSE113086 reviewer token: gjsjkqugbvsznut

Files in database submission

Raw Files:
MG_Smarca2MO_2_Input-116183131.R1_001.fastq.gz
MG_Smarca2MO_2_H3K9me3-116175180.R1_001.fastq.gz
MG_Smarca2MO_1_Input-116189140.R1_001.fastq.gz
MG_Smarca2MO_1_H3K9me3-116187136.R1_001.fastq.gz
MG_ScrambleMO_2_Input-116179142.R1_001.fastq.gz
MG_ScrambleMO_2_H3K9me3-116173211.R1_001.fastq.gz
MG_ScrambleMO_1_Input-116183127.R1_001.fastq.gz
MG_ScrambleMO_1_H3K9me3-116188134.R1_001.fastq.gz
MG_6_2_Input-116184127.R1_001.fastq.gz
MG_6_2_H3K9me3-116183126.R1_001.fastq.gz
MG_6_1_Input-116175183.R1_001.fastq.gz
MG_6_1_H3K9me3-116188129.R1_001.fastq.gz
MG_4_5_2_Input-116178178.R1_001.fastq.gz
MG_4_5_2_H3K9me3-116179145.R1_001.fastq.gz
MG_4_5_1_Input-116191097.R1_001.fastq.gz
MG_4_5_1_H3K9me3-116177188.R1_001.fastq.gz
MG_2_5_2_Input-116173213.R1_001.fastq.gz
MG_2_5_2_H3K9me3-116174194.R1_001.fastq.gz
MG_2_5_1_Input-116188128.R1_001.fastq.gz
MG_2_5_1_H3K9me3-116180169.R1_001.fastq.gz

Processed Files:
Sorted_SizedMG_Smarca2MO_2_Input-116183131.R1_001_trimmed.MarkedDups.bam.DupsR.tdf
Sorted_SizedMG_Smarca2MO_2_H3K9me3-116175180.R1_001_trimmed.fq.gz.MarkedDups.bam.DupsR.tdf
Sorted_SizedMG_Smarca2MO_1_Input-116189140.R1_001_trimmedMarkedDups.bam.DupsR.tdf
Sorted_SizedMG_Smarca2MO_1_H3K9me3-116187136.R1_001_trimmedMarkedDups.bam.DupsR.tdf
Sorted_SizedMG_ScrambleMO_2_Input-116179142.R1_001_trimmedMarkedDups.bam.DupsR.tdf
Sorted_SizedMG_ScrambleMO_2_H3K9me3-116173211.R1_001_trimmedMarkedDups.bam.DupsR.tdf
Sorted_SizedMG_ScrambleMO_1_Input-116183127.R1_001_trimmedMarkedDups.bam.DupsR.tdf
Sorted_SizedMG_ScrambleMO_1_H3K9me3-116188134.R1_001_trimmedMarkedDups.bam.DupsR.tdf
Sorted_SizedMG_6_2_Input-116184127.R1_001_trimmedMarkedDups.bam.DupsR.tdf
Sorted_SizedMG_6_2_H3K9me3-116183126.R1_001_trimmedMarkedDups.bam.DupsR.tdf
Sorted_SizedMG_6_1_Input-116175183.R1_001_trimmedMarkedDups.bam.DupsR.tdf
Sorted_SizedMG_6_1_H3K9me3-116188129.R1_001_trimmedMarkedDups.bam.DupsR.tdf
Sorted_SizedMG_4_5_2_Input-116178178.R1_001_trimmedMarkedDups.bam.DupsR.tdf

Sorted_SizedMG_4_5_2_H3K9me3-116179145.R1_001_trimmedMarkedDups.bam.DupsR.tdf
Sorted_SizedMG_4_5_1_Input-116191097.R1_001_trimmedMarkedDups.bam.DupsR.tdf
Sorted_SizedMG_4_5_1_H3K9me3-116177188.R1_001_trimmedMarkedDups.bam.DupsR.tdf
Sorted_SizedMG_2_5_2_Input-116173213.R1_001_trimmedMarkedDups.bam.DupsR.tdf
Sorted_SizedMG_2_5_2_H3K9me3-116174194.R1_001_trimmedMarkedDups.bam.DupsR.tdf
Sorted_SizedMG_2_5_1_Input-116188128.R1_001_trimmedMarkedDups.bam.DupsR.tdf
Sorted_SizedMG_2_5_1_H3K9me3-116180169.R1_001_trimmedMarkedDups.bam.DupsR.tdf

Genome browser session
(e.g. UCSC)

http://epigenome.genetics.uga.edu/SchmitzLab-JBrowse/?data=Goll_MZT_Seq

Methodology

Replicates

Two biological replicates were generated for ChIP-seq experiments

Sequencing depth

All sequencing runs were single-end generating 75 base pair reads. Below are the stats for the files that were trimmed of adapters and had duplicates marked. The tdf files in GEO had further processing to remove duplicates.
Sorted_SizedMG_Smarca2MO_2_Input-116183131.R1_001_trimmed.MarkedDups.bam
Total: 30838394 Duplicates: 3488630 Mapped: 23862658
Sorted_SizedMG_Smarca2MO_2_H3K9me3-116175180.R1_001_trimmed.fq.gz.MarkedDups.bam
Total: 18459227 Duplicates: 3057700 Mapped: 15108710
Sorted_SizedMG_Smarca2MO_1_Input-116189140.R1_001_trimmedMarkedDups.bam
Total: 21633102 Duplicates: 2673527 Mapped: 13463567
Sorted_SizedMG_Smarca2MO_1_H3K9me3-116187136.R1_001_trimmedMarkedDups.bam
Total: 14395299 Duplicates: 3880542 Mapped: 11895054
Sorted_SizedMG_ScrambleMO_2_Input-116179142.R1_001_trimmedMarkedDups.bam
Total: 27494966 Duplicates: 2787094 Mapped: 15853872
Sorted_SizedMG_ScrambleMO_2_H3K9me3-116173211.R1_001_trimmedMarkedDups.bam
Total: 16791888 Duplicates: 7745869 Mapped: 12533367
Sorted_SizedMG_ScrambleMO_1_Input-116183127.R1_001_trimmedMarkedDups.bam
Total: 31219429 Duplicates: 7423596 Mapped: 23901507
Sorted_SizedMG_ScrambleMO_1_H3K9me3-116188134.R1_001_trimmedMarkedDups.bam
Total: 22167287 Duplicates: 11807896 Mapped: 17832612
Sorted_SizedMG_6_2_Input-116184127.R1_001_trimmedMarkedDups.bam.DupsR.bam
Total: 52692030 Duplicates: 2514756 Mapped: 45357715
Sorted_SizedMG_6_2_H3K9me3-116183126.R1_001_trimmedMarkedDups.bam
Total: 20846231 Duplicates: 1911630 Mapped: 17854837
Sorted_SizedMG_6_1_Input-116175183.R1_001_trimmedMarkedDups.bam
Total: 35727600 Duplicates: 1381493 Mapped: 30217493
Sorted_SizedMG_6_1_H3K9me3-116188129.R1_001_trimmedMarkedDups.bam
Total: 32937945 Duplicates: 3473873 Mapped: 28189308
Sorted_SizedMG_4_5_2_Input-116178178.R1_001_trimmedMarkedDups.bam
Total: 21973923 Duplicates: 811876 Mapped: 18877840
Sorted_SizedMG_4_5_2_H3K9me3-116179145.R1_001_trimmedMarkedDups.bam
Total: 14333553 Duplicates: 1042291 Mapped: 12205470
Sorted_SizedMG_4_5_1_Input-116191097.R1_001_trimmedMarkedDups.bam
Total: 22728671 Duplicates: 886940 Mapped: 19470036
Sorted_SizedMG_4_5_1_H3K9me3-116177188.R1_001_trimmedMarkedDups.bam
Total: 13846235 Duplicates: 861128 Mapped: 11839681
Sorted_SizedMG_2_5_2_Input-116173213.R1_001_trimmedMarkedDups.bam
Total: 15120921 Duplicates: 1204632 Mapped: 8995099
Sorted_SizedMG_2_5_2_H3K9me3-116174194.R1_001_trimmedMarkedDups.bam
Total: 11807768 Duplicates: 2712830 Mapped: 6414009
Sorted_SizedMG_2_5_1_Input-116188128.R1_001_trimmedMarkedDups.bam
Total: 16754772 Duplicates: 1107885 Mapped: 10131442
Sorted_SizedMG_2_5_1_H3K9me3-116180169.R1_001_trimmedMarkedDups.bam
Total: 6457373 Duplicates: 573904 Mapped: 5023906

Antibodies

anti-H3K9me3antibody (abcam ab8898)

Peak calling parameters

As described in materials and methods, aligned reads (.bam format) were used to create HOMER tag directories. Peaks were called using the HOMER findPeaks software package. Relevant parameters are: -style histone -size 1000. The default parameter for -minDist was used(1000bp). Thus, adjacent peaks that were less than 1000bp apart were merged by HOMER software to create a single peak.

Data quality

TrimGalore/cutadapt/fastqc were used to trim any adapters or sequences below 20bp. Duplicates were marked using Picard tools and removed using samtools.
All peaks called had a minimum fold change of 4 or higher relative to the input sample

In the 6hpf Merged dataset, 17,549 peaks have a p-value <0.05
In the 6hpf Merged dataset, 3,354 peaks have a fold change of >=5

In the Smarca2 MO Merged dataset, 12,748 peaks have a p-value <0.05
In the Smarca2 MO Merged dataset, 4,249 peaks have a fold change of >=5

Software

Libraries pooled/sequenced on a NextSeq500 instrument at the Georgia Genomics Facility. Short reads (<20bp) and adapter sequences were removed using TrimGalore, cutadapt, and Python with fastqc command. Trimmed Illumina reads aligned to Zebrafish genome assembly (Accession # GCF_000002035.6) using BWA algorithms aln, and samse, which randomly assign multiply mapped reads to a single location. Duplicate reads were marked and removed using Picard tools and samtools. To plot the relative distribution of mapped reads, read counts were determined using igvtools and data were displayed using IGV. HOMER was used to identify H3K9me3 peaks in 6 hpf and smarca2 knockdown embryos using "findPeaks" -style histone -size 1000. Bedtools "intersect" was used compare peak locations between samples. HOMER was also used to construct metaplots or heatmaps centered on the H3K9me3 peaks identified in the combined 6 hpf sequence data using "annotatePeaks.pl" -hist 50 -size 10000 and with or without the -ghist flag to make heatmaps and metaplots, respectively. Heatmaps were constructed with R using the pheatmap package.
Sequence data are available through the NCBI GEO database (accession # GSE113086; secure reviewer token: gjsjkqugbvsznut).
