# Supplementary material for: The maternal to zygotic transition regulates genome-wide heterochromatin establishment in the zebrafish embryo
Source: Nat Commun. 2019 Apr 4;10:1551. doi: 10.1038/s41467-019-09582-3 (PMC6449393; doi:10.1038/s41467-019-09582-3)
Supplement: Supplementary file 4 — Source Data [file 41467_2019_9582_MOESM4_ESM.zip › sourcedata.pdf]

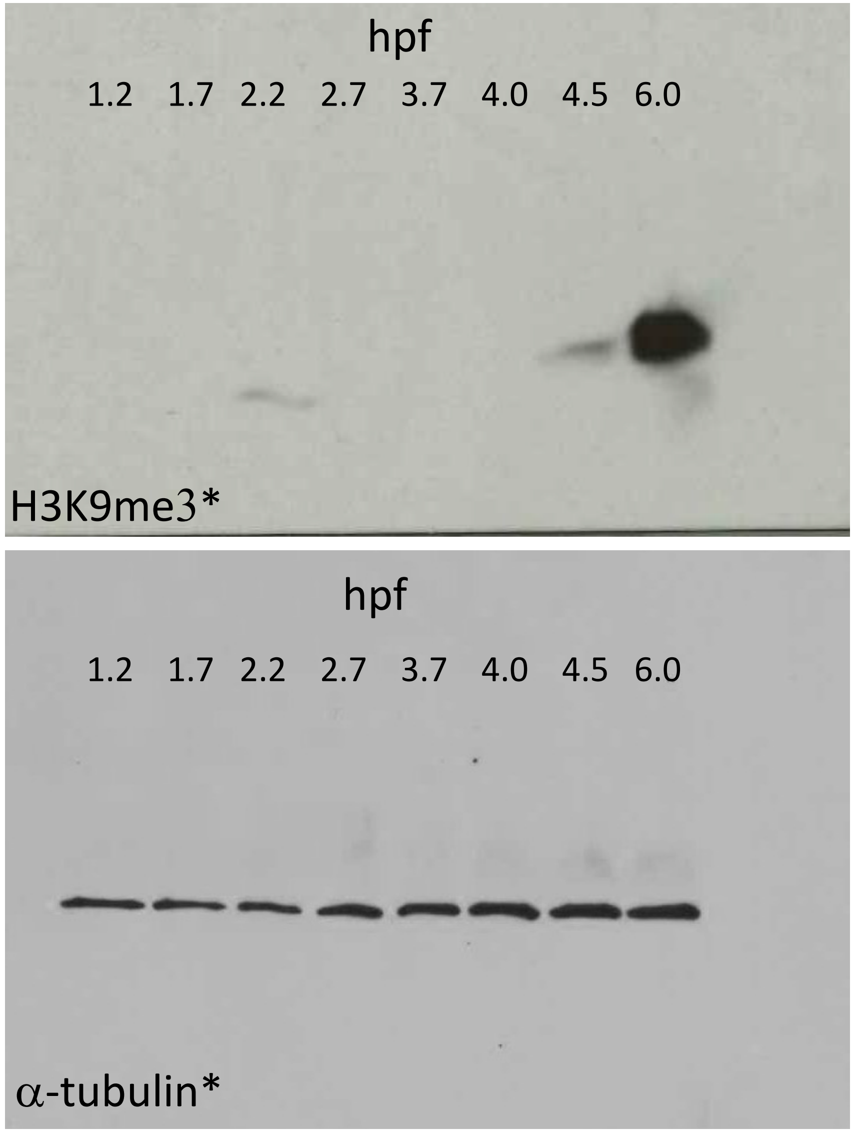

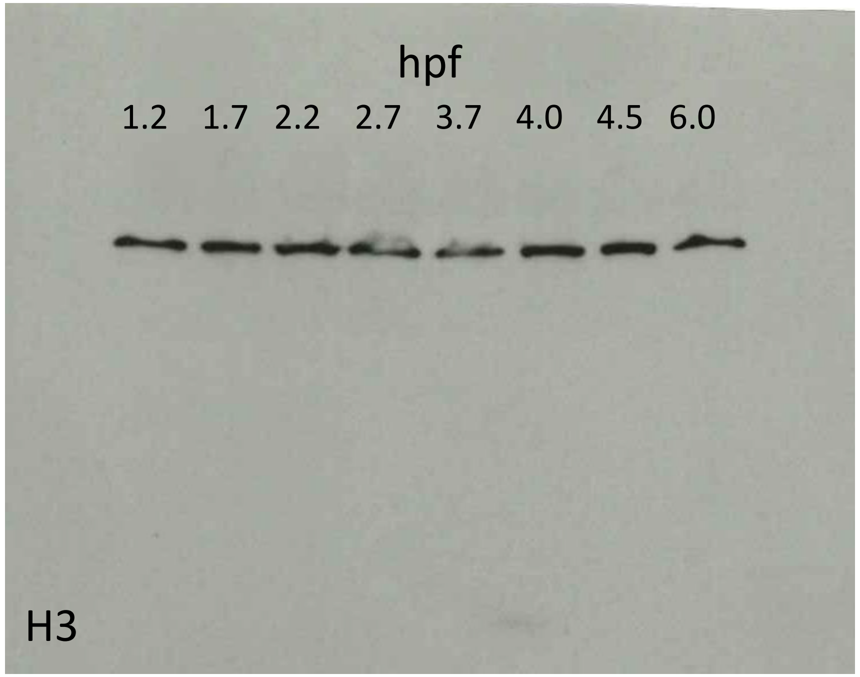

*represent the same membrane, cut and separately probed with α-tubulin or H3K9me3 antibody. H3 represents same samples loaded on separate gel.

Figure 1c

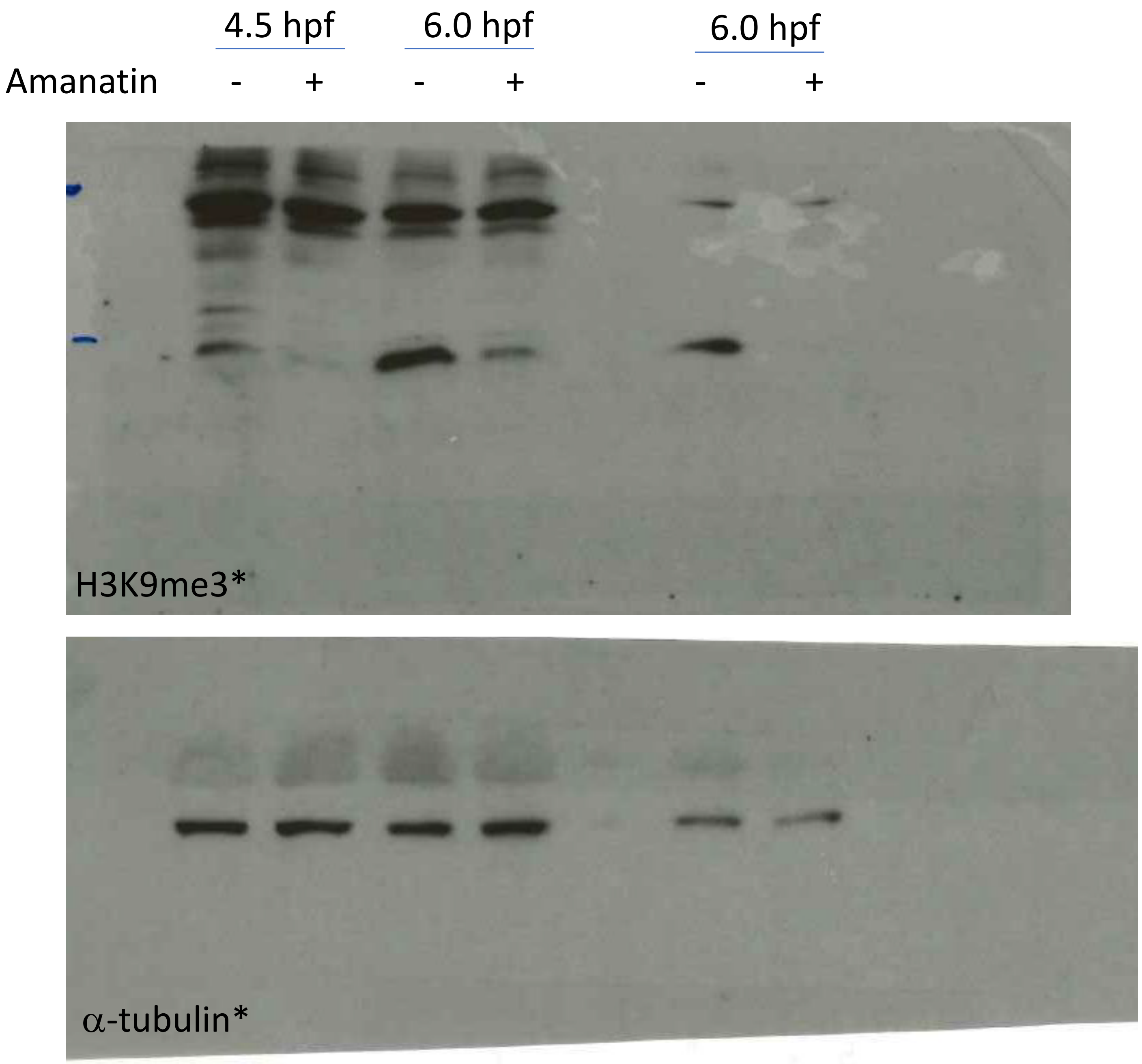

Figure 3a

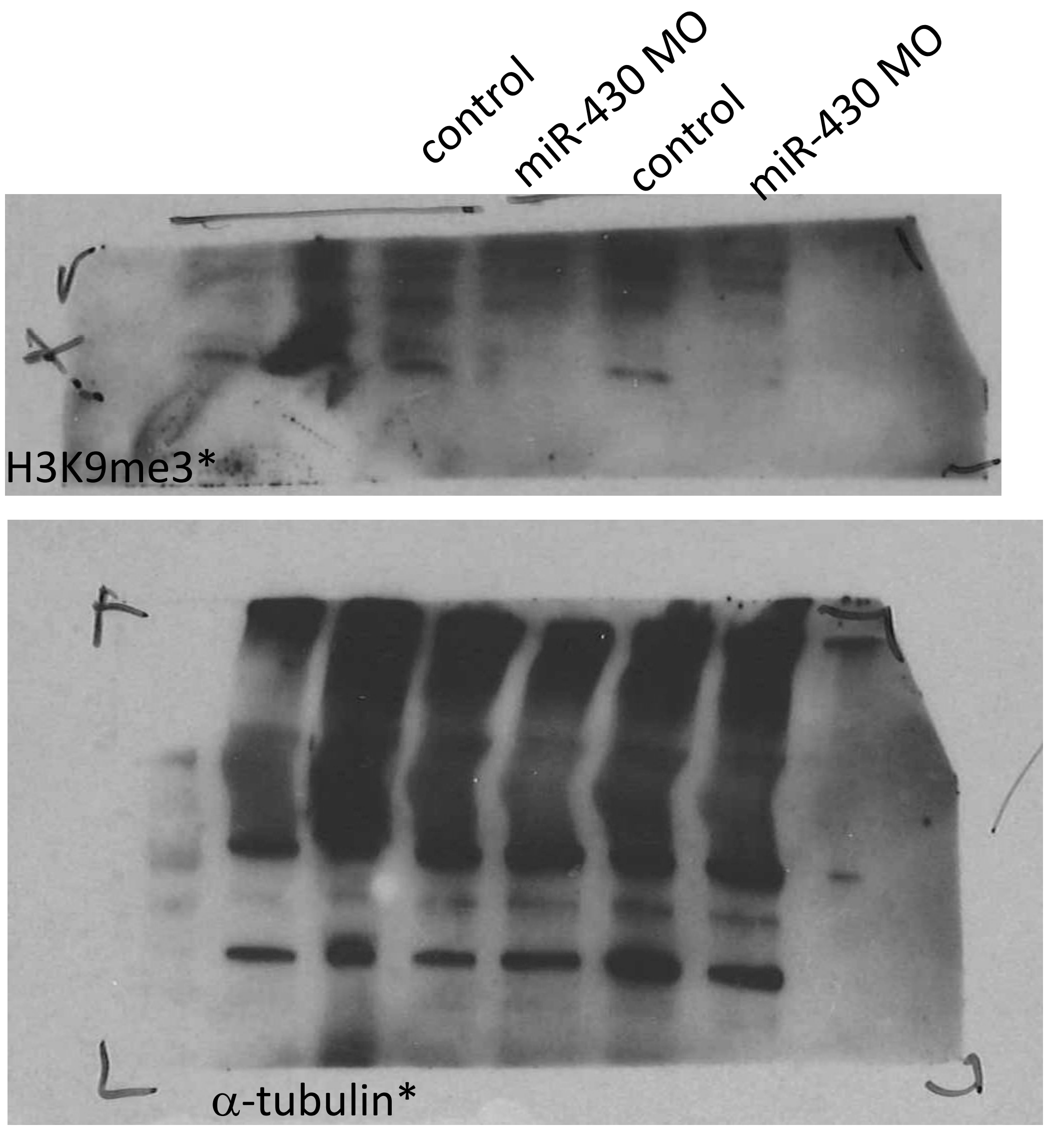

Figure 4a

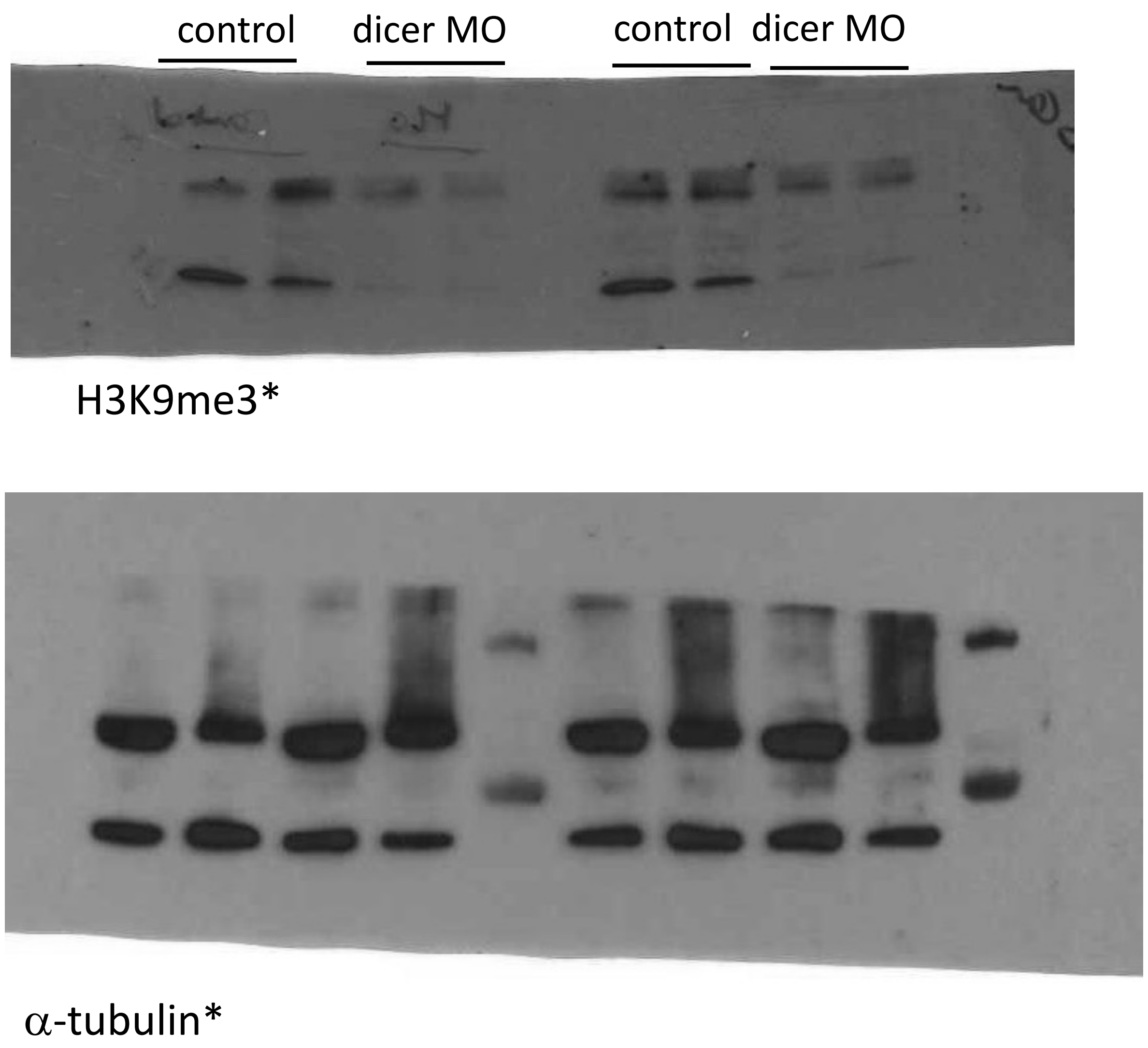

Figure 4b

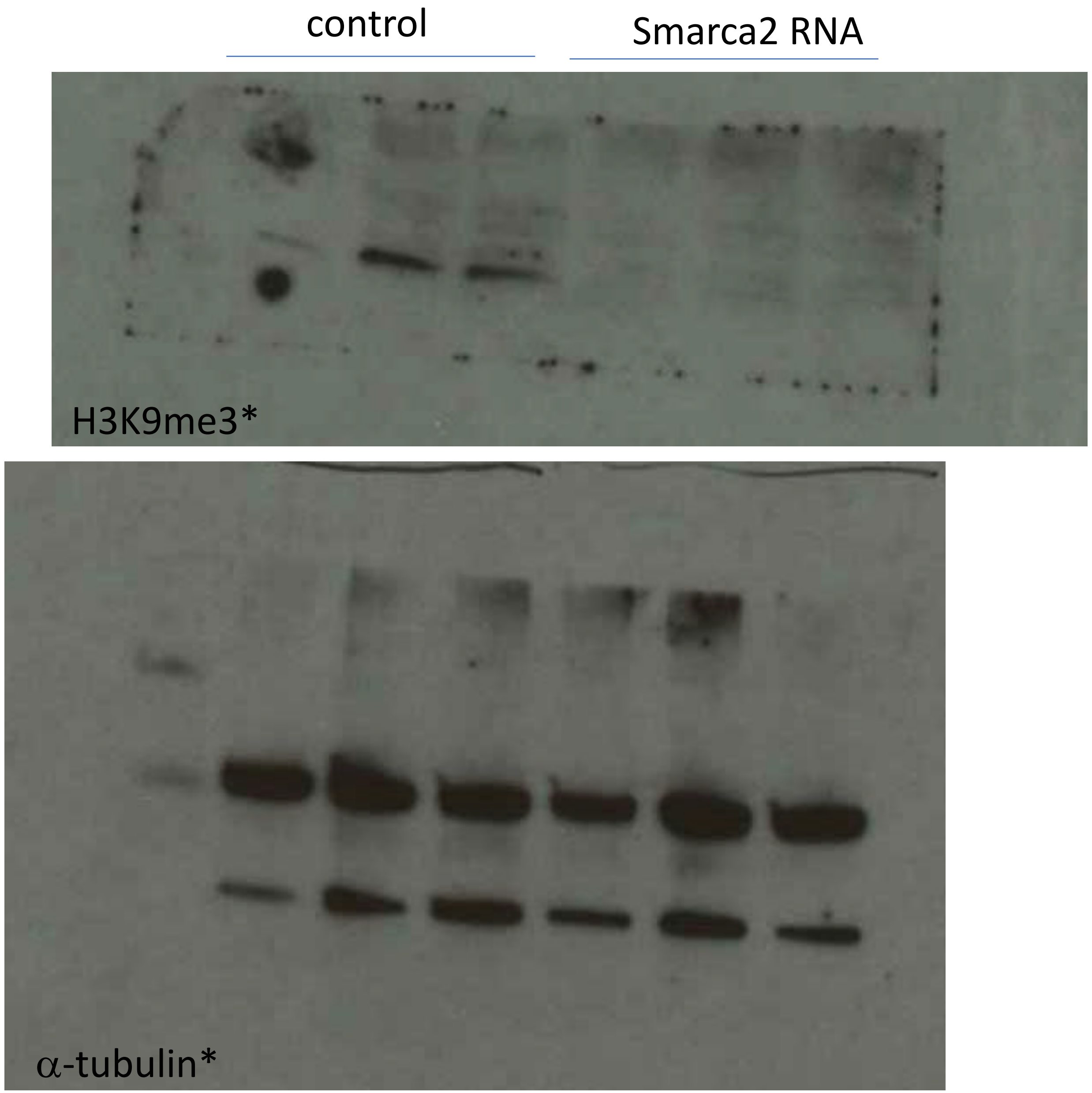

Figure 5c

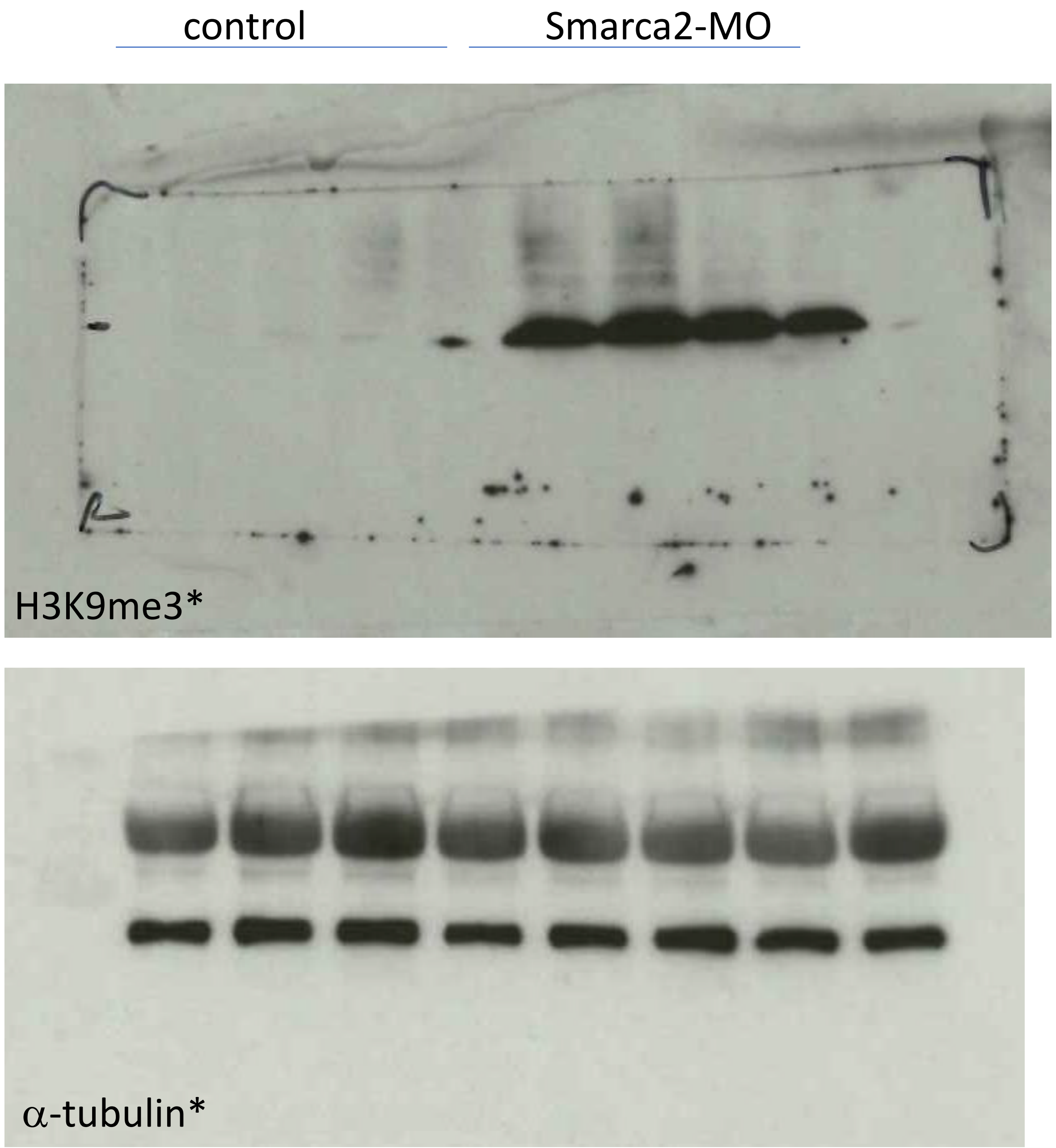

Figure 5d

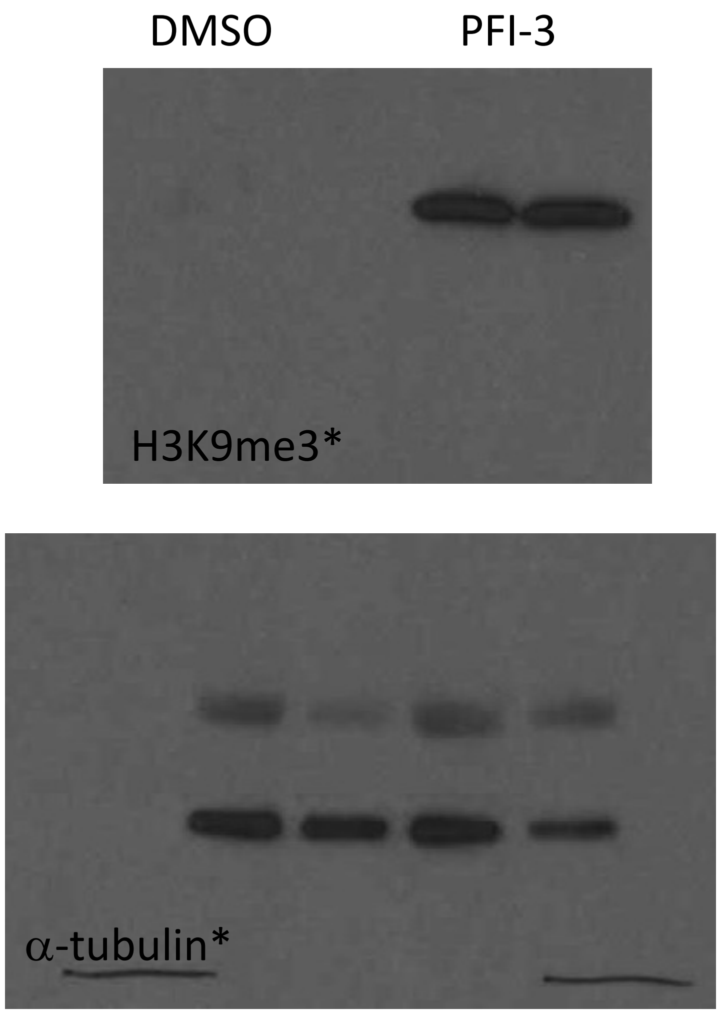

Fig 6a

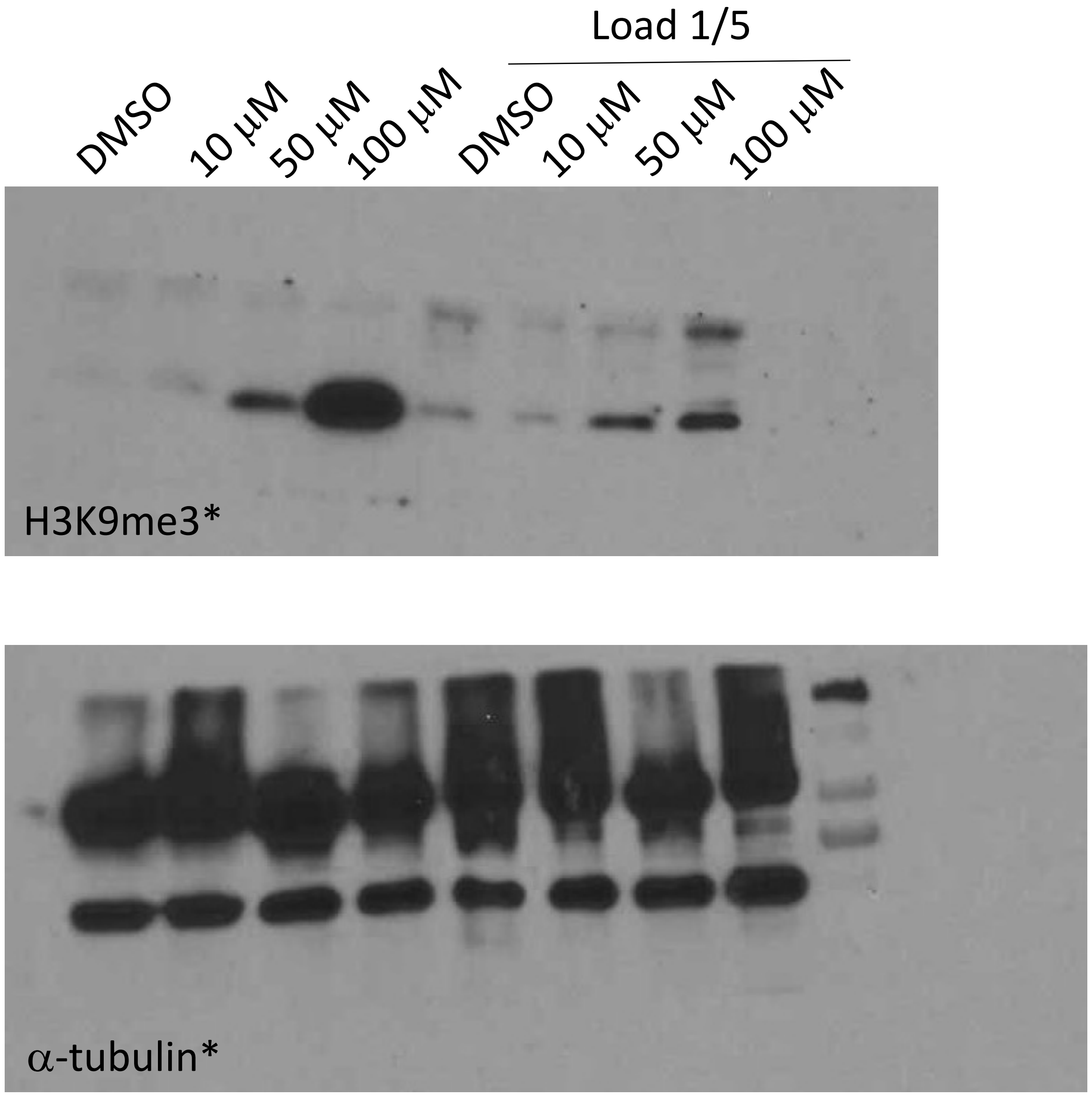
Load 1/5
DMSO
10 μM
50 μM
100 μM
DMSO
10 μM
50 μM
100 μM
H3K9me3*
α-tubulin*

Fig 6b
