# Supplementary material for: The maternal to zygotic transition regulates genome-wide heterochromatin establishment in the zebrafish embryo
Source: Nat Commun. 2019 Apr 4;10:1551. doi: 10.1038/s41467-019-09582-3 (PMC6449393; doi:10.1038/s41467-019-09582-3)
Supplement: Supplementary file 2 — Peer Review File [file 41467_2019_9582_MOESM2_ESM.pdf]

Reviewers' comments:

Reviewer #1 (Remarks to the Author):

In “The maternal to zygotic transition regulates genome-wide heterochromatin establishment in the zebrafish embryo” by Laue et al., the authors describe the establishment of heterochromatin during zebrafish embryogenesis. The work shows that upon fertilization, the presence of Smarca2 prevents heterochromatin formation. Upon the transcription of miR-430, Smarca2 RNA is degraded and heterochromatin is formed. This mechanism ensures the presence of an open chromatin structure until genome activation.

The paper is very well-written and clear. The data is interesting and supports (most of the) conclusions. I only have a few concerns, that I listed below

MAJOR CONCERNS
I have a problem with the interpretation of the data presented in Figure 3. The inhibition of transcription results in an arrest of zebrafish embryos at sphere stage. This means that there cannot be a comparison of “stage-matched” embryos as indicated in the text. The embryos can be “time-matched” but that is something else. I therefore think that the conclusion that “genome activation is required for heterochromatin formation” is not supported by the data. Obviously, there is evidence for this point later (if miR-430 is required, then transcription is required) but at this point in the manuscript I don't think this conclusion can be drawn yet.

MINOR CONCERNS
General
The Western blots that have been performed cannot be quantified because they are not quantitative. This applies to Figure 6B, Extended Figure 1, Extended Figure 3A, C, E, and Extended Figure 4B-D.

Figure 1
B. No indication of what scale bar represents.
C. I assume that the same number of embryos was used in all lanes? Mention this?
E. Perhaps indicate that signal is centered on peak center?

Figure 2
E-H. No indication of what scale bar represents.
J, K. How many nuclei/embryos were analyzed?

Figure 3.
C. No indication of what scale bar represents.
D,E. I would think that for reproducibility more than one embryo should be analyzed? Same in Figure 2 if that was only one embryo per stage?
In legends there is an (i) too much?

Figure 4.
A, B. How was the effectiveness of the miR-430 and Dicer MO tested?

Figure 6
C. No indication of what scale bar represents.
D. I would think that for reproducibility more than one embryo should be analyzed?

Extended 2
A. How should we interpret the difference between the two replicates at 6hpf?

Line
134-136. Transcript levels are not just comparable, they might even be reduced?
177. Figure reference incorrect?
483. And protein?

SUGGESTIONS
Would a more interesting title not focus on Smarca2 rather than the more general MZT?

In Figure 7, it would be clearer I think if the transcription of miR-430 is shown as transcription (preceding the canonical onset of transcription at 3hpf).

Reviewer #2 (Remarks to the Author):

This is a nice story from Mary Goll and her lab about the onset of heterochromatin formation during development. The authors combine genomics, electron microscopy and biochemistry to show that heterochromatin and H3K9me3 accumulate slowly, after the 1000 cell stage, and require i) zygotic transcription and ii) loss of smarca2. Together, these allow temporal regulation of heterochromatin onset. I liked the paper for its clear analysis but there are two critical issues and one confusing one:

- The authors rely on morpholinos, which can generate artifacts. There is certainly a mutant for Dicer that could be obtained and studied, and miR-430 could be added back to Dicer mutants as a morpholino independent assay. A mutant for smarca2 would also be very helpful. Relying only on morpholinos can lead one astray.

- How do the authors control for TEM variability? Can they ensure that other structures are normal in treated vs control embryos?

- Finally, there is considerable H3K9me3 left after alpha amanitin treatment (circa 50%). Why? Does this mean there are two forms of H3K9me3 deposition, one sensitive to transcription and one independent? Can the authors determine how well transcription has been inactivated? This experiment should also be tidied up. If this result is solid, the authors should rewrite their text to reflect the partial effects

Reviewer #3 (Remarks to the Author):

Laue et al. The maternal to zygotic transition regulates genome-wide heterochromatin establishment in the zebrafish embryo. This manuscript demonstrates very clearly the delayed onset of heterochromatin establishment in the developing zebrafish embryo and uncovers the mechanistic basis controlling this at the molecular level. The results are clearly presented and appropriately interpreted. My specific comments are detailed below:

1. Fig. 1b The N numbers are not presented. At 3.7 hpf for example how many embryos show the depicted staining pattern?

2. Fig 1 d-f. A deeper analysis of the H3K9me3 Chipseq dataset would be advisable. The authors mention that at 6hpf, approx. 80% of K9me3 called peaks overlapping annotated repeats. Which repeat families enriched? What is the remaining 20%? Genes or intergenic regions? Are there any gene ontology terms enriched for K9me3?

3. Normalization of qPCR data is difficult during early stages of embryogenesis due to the genome-wide changes in transcript abundance. Is 18S alone a suitable normalizing reference? Is its expression constant across all stages? Applies to Extended Data 1b-e and Fig 5a.
4. The authors conclude that H3K9me3 is sufficient for heterochromatin establishment during zebrafish embryogenesis. However the assessment of heterochromatin establishment is based purely on the chromatin compaction assessed by electron microscopy. While this is a very interesting point I would prefer to see another readout in addition to electron microscopy. Can the authors assess other markers of constitutive heterochromatin, such as H4K20me3 or H3K64me3, to determine whether they follow H3K9me3 during normal embryogenesis, and whether they are precociously acquired by Smarca2-MO? Alternatively the authors could assess chromocentre formation in this context, independently of H3K9me3 staining.

5. Figure 5. To what extent do ectopically gained H3K9me3 peaks in Smarca4 knockdown embryos overlap with endogenous peaks at 4.5 or 6.0 hpf?
6. Figure 3. Remove i) in the legend

7. The authors could go further with this work, having uncovered a clear model in which heterochromatin establishment is established ectopically early, whether this affects development of the animal? So, for example to test development of the embryos after Smarca2-MO or PFI-3 injection. This will address the physiological relevance of the authors' finding of delayed heterochromatin establishment in the early zebrafish embryo.

Reviewer response:

We are grateful for reviewer comments and for the opportunity to submit a revised version of our manuscript. We were especially pleased to read that overall, reviewers felt the paper was well written with clear analysis, and that the data were interesting and appropriately interpreted. We respond to individual comments from reviewers below, and have marked new text in the revised manuscript in red to highlight changes outlined in our responses.

Reviewers' comments:

Reviewer #1 (Remarks to the Author):

In “The maternal to zygotic transition regulates genome-wide heterochromatin establishment in the zebrafish embryo” by Laue et al., the authors describe the establishment of heterochromatin during zebrafish embryogenesis. The work shows that upon fertilization, the presence of Smarca2 prevents heterochromatin formation. Upon the transcription of miR-430, Smarca2 RNA is degraded and heterochromatin is formed. This mechanism ensures the presence of an open chromatin structure until genome activation.

The paper is very well-written and clear. The data is interesting and supports (most of the) conclusions. I only have a few concerns, that I listed below

MAJOR CONCERNS
I have a problem with the interpretation of the data presented in Figure 3. The inhibition of transcription results in an arrest of zebrafish embryos at sphere stage. This means that there cannot be a comparison of “stage-matched” embryos as indicated in the text. The embryos can be “time-matched” but that is something else. I therefore think that the conclusion that “genome activation is required for heterochromatin formation” is not supported by the data. Obviously, there is evidence for this point later (if miR-430 is required, then transcription is required) but at this point in the manuscript I don't think this conclusion can be drawn yet.

We agree with this reviewer concern, and regret not being more careful with our language. We typically perform our experiments with time and stage matched controls. However, in this case, only analysis of time matched embryos was possible when α-amanatin concentrations that completely block ZGA were used. We very much thank the reviewer for catching the error in the description of our experimental approach, and we have been careful to verify that similar errors were not included elsewhere in the manuscript. Embryos injected with α-amanatin continue cell division until they die at 8-10 hours post fertilization (hpf), but are reported to arrest normal development between 4 and 4.5 hpf and do not undergo gastrulation (Lee et al Nature 2013, Kane et al Development 1996). To partially deal with this issue, analysis was first performed at 4.5 hpf. At this stage, we found that mock and α-amanatin injected embryos were still morphologically quite similar but had reduced H3K9me3. Given that concerns associated with the 6 hpf time point were raised by both Reviewer 1 and Reviewer 2, and that this data point is not critical for any of our conclusions, we have opted to remove it from the manuscript.

We also agree that while additional experiments in this manuscript support a role for zygotic genome activation in regulating heterochromatin through transcription of miR-430, in this particular experiment even at 4.5 hpf, we can only say that blocking transcription reduced H3K9me3/ chromatin compaction. This experiment does not allow us to clearly assess whether this reduction reflects a direct requirement for zygotic genome activation in heterochromatin

regulation, or a more indirect effect associated with downstream consequences of blocking transcription. We have amended our language to be more precise and to better reflect this caveat.

MINOR CONCERNS

General

The Western blots that have been performed cannot be quantified because they are not quantitative. This applies to Figure 6B, Extended Figure 1, Extended Figure 3A, C, E, and Extended Figure 4B-D.

We again apologize for the lack of precision in our language. We agree that Westerns are not truly quantitative and no longer use the word quantify in conjunction with these results. However, we do find that when developing our films below a saturating exposure and examining both H3K9me3 and α-tubulin from the same blot at the same time, the ratios of H3K9me3 to a-tubulin are similar across similar experimental samples. We feel that there is value in demonstrating that our results are consistent across many samples. Therefore, we still include these analysis in extended data. However, we are now more careful in our description of the results. We now also include primary western data for PFI-3 dosage response in panel 6b.

Figure 1

B. No indication of what scale bar represents.

We apologize for this omission and now indicate that the scale bar represents 1μM in the legend for Fig1 b.

C. I assume that the same number of embryos was used in all lanes? Mention this?

We now indicate that all samples were prepared from 20 embryos with 1/3 of each protein lysate loaded into the corresponding lane in the figure legend for 1C.

E. Perhaps indicate that signal is centered on peak center?

We now indicate that the signal is centered on peak center in the figure legend for Fig 1e

Figure 2

E-H. No indication of what scale bar represents.

We apologize for this omission and now indicate that the scale bar for Fig 2a-h represents 1μM in the corresponding legend.

J, K. How many nuclei/embryos were analyzed?

We have broadly examined at least 5 embryos for each time point with general scanning suggesting that that our imaging and quantification is reflective of a multitude of nuclei in multiple embryos at each stage. Quantitative analysis in Fig 2 j-k represents combined data from 3 embryos per time point with 4-6 representative nuclei examined from each embryo. Each

dot on graphs in j and k represents data from one nucleus. We have revised the figure legend for Fig 2 to make this clearer.

Figure 3.
C. No indication of what scale bar represents.

We apologize for this omission and now indicate that the scale bar for Fig 3C represents 1μM in the corresponding legend.

D,E. I would think that for reproducibility more than one embryo should be analyzed? Same in Figure 2 if that was only one embryo per stage?

We apologize that this wasn't clear. In Figure 3 each data point represents an embryo, with data for 6-10 nuclei averaged to generate the value for each embryo. In total, we report data on 4 wild type and 4 α–amanatin injected embryos. We have now revised the legend for Fig 3 to make this more clear

As indicated above, nuclei from three embryos per stage were assessed in Fig 2.

In legends there is an (i) too much? Thank you for catching this mistake, we have removed.

Figure 4.
A, B. How was the effectiveness of the miR-430 and Dicer MO tested?

The Dicer and miR-430 MOs used in this report have been previously published, and we now include these references in our methods. Effectiveness of the morpholinos in our hands was demonstrated by sustained expression of transcripts that are normally degraded by the dicer/miR430 pathway at MZT including gstm, pfn2 and aldh7a1. We now include expression data for these genes in Extended data 4.

Figure 6
C. No indication of what scale bar represents.

We apologize for this omission and now include that the scale bar represents 1μM in the figure legend for Fig 6C

D. I would think that for reproducibility more than one embryo should be analyzed?

We apologize that this wasn't clear. Each data point on graphs in panel 6d indicates an individual embryo. Four DMSO and nine PFI-3 injected embryos were examined in total. For each embryo values for particles per um^2 and percent nuclear area reflect average values derived from 5-10 representative nuclei. We have now added additional information to the legend text which makes our approach clearer.

Extended 2
A. How should we interpret the difference between the two replicates at 6hpf?

The difference between the two 6 hpf samples are likely to reflect differences in ChIP efficiencies. Alternatively, this difference could reflect subtle differences in developmental stage between the two pools of embryos. We now mention these possibilities in the legend associated with Extended data 1b. To confirm the relatedness of samples we have now performed additional peak calling and sample clustering using the Chip-dif. This data reveals that H3K9me3 peaks in the two 6 hpf samples are more similar to each other than to H3K9me3 peaks called in samples from other time points. See Extended data 1D.

Line
134-136. Transcript levels are not just comparable, they might even be reduced?

We agree and have amended the text to state this.

177. Figure reference incorrect?

We have deleted this extraneous figure reference.

483. And protein?

We now state that "The early embryo relies exclusively on maternally deposited RNA transcripts <u>and protein</u>." We agree this is an important edit and thank the reviewer for suggesting it.

SUGGESTIONS
Would a more interesting title not focus on Smarca2 rather than the more general MZT?

We thank the reviewer for this suggestion, it reflects something we thought about extensively. We agree a title focused on Smarca2 could also be appropriate. However, after consulting many colleagues, the consensus was that a title emphasizing the link between MZT and developmental control of when heterochromatin is established would better highlight the conceptual advances of the paper and would likely appeal to a broader audience.

In Figure 7, it would be clearer I think if the transcription of miR-430 is shown as transcription (preceding the canonical onset of transcription at 3hpf).
We have amended our figure to include a separate line for miR430

Reviewer #2 (Remarks to the Author):

This is a nice story from Mary Goll and her lab about the onset of heterochromatin formation during development. The authors combine genomics, electron microscopy and biochemistry to show that heterochromatin and H3K9me3 accumulate slowly, after the 1000 cell stage, and

require i) zygotic transcription and ii) loss of smarca2. Together, these allow temporal regulation of heterochromatin onset. I liked the paper for its clear analysis but there are two critical issues and one confusing one:

- The authors rely on morpholinos, which can generate artifacts. There is certainly a mutant for Dicer that could be obtained and studied, and miR-430 could be added back to Dicer mutants as a morpholino independent assay. A mutant for smarca2 would also be very helpful. Relying only on morpholinos can lead one astray.

We agree with the reviewer that morpholinos must be used carefully in experimental settings and that where possible, additional analysis using secondary approaches can provide important additional support for morpholino based findings. However, it is also important to realize that many earlier reports of discrepancies between morpholino and mutant phenotypes such as (Kok et al Dev Cell, 2015) are now thought to reflect a phenomenon termed transcriptional compensation rather than morpholino artifacts as first proposed (El-Brolosy and Stainier Plos Genetics 2017, Rossi et al Nature 2015, bioRxiv 328153; doi: https://doi.org/10.1101/328153).

In our case, use of mutants is complicated by maternal RNA deposition of *dicer/ smarca2*. This means that in both cases, zygotic mutants generated from crossing homozygous adults will still have maternally derived RNA/protein for these genes at stages that are relevant for our analysis. Because homozygous *dicer* mutants are lethal before adulthood (and our morpholino data suggests the same will be true for *smarca2* mutants), we cannot simply generate maternal/zygotic mutant embryos by crossing homozygous mutant adults. Although theoretically feasible, our lab has not been able to generate wildtype adults with homozygous mutant germlines for either of these genes by germline transplantation or directed deletion of these genes in the germline using targeted Crispr/cas9. We have also been unable to identify any laboratories that currently have maternal zygotic dicer animals which could be used for such analysis. Instead, we have performed experiments that are consisted with existing standards in the zebrafish field for morpholino use when mutant analysis is not possible. These include use of more than one morpholino targeting a gene or gene pathway, recapitulation of similar phenotypes with chemical inhibitors and demonstration of a reciprocal phenotype by overexpression of the relevant RNA.

In the current manuscript, the dicer morpholino was used as a control to show that depletion of a factor required for miR430 biogenesis recapitulated phenotypes seen with the miR430 morpholino, thereby demonstrating that depletion of two genes in the same pathway generated similar phenotypes. As suggested by the reviewer, we attempted the additional control of rescuing H3K9me3 in dicer morphants through coinjection of synthetic miR430 RNA duplexes. However, we found that injection of these duplexes at the 1 cell stage impaired embryonic development prior to zygotic genome activation, making analysis difficult. Given that we have demonstrated delay of H3K9me3 establishment with morpholinos targeting two different genes in the miR-430 pathway, that previous published results demonstrate that Smarca2 is a bonafide target of miR430 mediated degradation at MZT, and that our subsequent analysis demonstrates downregulation of Smarca2 at MZT is required for H3K9me3 establishment, we feel the main conclusions of this manuscript are well supported even without this additional control.

For Smarca2, we demonstrate that two different morpholinos accelerate the timeline of H3K9me3 establishment and that this phenotype is recapitulated by chemical inhibition of

Smarca2 in a dose dependent manner. Conversely, we demonstrate that injection of mRNA encoding Smarca2 delays H3K9me3 incorporation. Hence our Smarca2 findings do not rely only on morpholinos, as chemical inhibitor and mRNA injection experiments provide two morpholino independent approaches

- How do the authors control for TEM variability? Can they ensure that other structures are normal in treated vs control embryos?

TEM images were captured using constant conditions and we controlled for illumination bias using previously published Image J plug-in designed for this correction which we cite in our methods (Boskovic et al 2014). All images were visually inspected to assure other cytoplasmic features appeared normal in images. We now include additional representative images in Extended data 1 which include surrounding cytoplasmic structures.

- Finally, there is considerable H3K9me3 left after alpha amanitin treatment (circa 50%). Why? Does this mean there are two forms of H3K9me3 deposition, one sensitive to transcription and one independent? Can the authors determine how well transcription has been inactivated? This experiment should also be tidied up. If this result is solid, the authors should rewrite their text to reflect the partial effects

Extended data Fig 3 does show a range of effectiveness for α–amanatin across samples in western blot data, and we agree that in Fig 3b, there appears to be some residual H3K9me3 signal at pericentromeres in α–amanatin injected embryos. These are complicated experiments because injection of α–amanatin can lead to embryo arrest (as raised by reviewer 1), and variability in injection across embryos can result in variable blocking of inhibition of ZGA in any given embryo. It is also not uncommon to observe some background signal in directed ChIP experiments. We do not have a way to simultaneously assess blockage of ZGA and H3K9me3 levels in the same embryo. In the interest of transparency, we felt it appropriate to included data from all experiments rather than only selecting those which showed the strongest effect. Because we can't rigorously demonstrate that residual H3K9me3 in some experiments is due to partial blockage of ZGA, we are careful to only make the claim that blocking ZGA impairs H3K9me3 establishment in the text. To further improve clarity on this point, we now comment on the variability of the effect in the legend for extended data figure 3.

Reviewer #3 (Remarks to the Author):

Laue et al. The maternal to zygotic transition regulates genome-wide heterochromatin establishment in the zebrafish embryo. This manuscript demonstrates very clearly the delayed onset of heterochromatin establishment in the developing zebrafish embryo and uncovers the mechanistic basis controlling this at the molecular level. The results are clearly presented and appropriately interpreted. My specific comments are detailed below:.

1. Fig. 1b The N numbers are not presented. At 3.7 hpf for example how many embryos show the depicted staining pattern?

For each time point at least 15 embryos were visually examined and images are representative of what we observed in 3 independent experiments. Imaged nuclei are representative of all observed nuclei within the whole mounted embryos. We now include this information in the figure legend for Fig 1b.

2. Fig 1 d-f. A deeper analysis of the H3K9me3 Chipseq dataset would be advisable. The authors mention that at 6hpf, approx. 80% of K9me3 called peaks overlapping annotated repeats. Which repeat families enriched? What is the remaining 20%? Genes or intergenic regions? Are there any gene ontology terms enriched for K9me3?

We thank the reviewer for this suggestion. We carried out additional bioinformatic analysis using a more recently generated repeat annotation, which revealed even more extensive overlap between early H3K9me3 peaks and repeated loci (96% of repeats). A figure depicting the overlap between H3K9me3 in various types of repeated sequences is now shown in Extended data 1e. We also analyzed the 4% of H3K9me3 peaks that showed no overlap with annotated repeats and found that these peaks most commonly occur in intergenic regions. The text has been modified to include a description of this new analysis.

3. Normalization of qPCR data is difficult during early stages of embryogenesis due to the genome-wide changes in transcript abundance. Is 18S alone a suitable normalizing reference? Is its expression constant across all stages? Applies to Extended Data 1b-e and Fig 5a.

We chose 18S for normalization based on McCurley and Callard (2008), which examined expression of a number of housekeeping genes across zebrafish development. This paper showed that 18S is constant at the stages that are relevant for our analysis, whereas other housekeeping genes exhibit more variable expression. Findings in this publication are consistent with our own analysis based on qRTPCR. Additional confidence in our results is derived from that fact that expression data reported in Fig 5a is consistent with the temporal expression patterns of Smarca2 in RNAseq data sets published by Harvey et al (2013) and White et al. (2017). We now cite McCurely and Callard in our methods, and include a graph of Smarca2 expression based on RNA-seq data from White et al 2017 in extended data 5a.

4. The authors conclude that H3K9me3 is sufficient for heterochromatin establishment during zebrafish embryogenesis. However, the assessment of heterochromatin establishment is based purely on the chromatin compaction assessed by electron microscopy. While this is a very interesting point I would prefer to see another readout in addition to electron microscopy. Can the authors assess other markers of constitutive heterochromatin, such as H4K20me3 or H3K64me3, to determine whether they follow H3K9me3 during normal embryogenesis, and whether they are precociously acquired by Smarca2-MO? Alternatively, the authors could assess chromocentre formation in this context, independently of H3K9me3 staining.

We made the statement that H3K9me3 is sufficient for heterochromatin establishment based on the definition of heterochromatin as condensed chromatin, which we felt was clearly visible in EMs. Interestingly, current data indicates that H4K20me3 and H3K64me3 are not associated with heterochromatin until after implantation in mouse, suggesting they may mark a more mature form of somatic heterochromatin (Wongtawan et al 2011, Eid et al 2016). Similarly, we are unable to detect H4K20me3 or H3K64me3 in wildtype zebrafish embryos until 8 hpf or later.

In zebrafish, clear chromocenters can be visualized by H3K9me3 antibody staining from about 4.5 hpf onward, however these chromocenters are not visible by DAPI at this stage or at any later stage of development that we have examined. Given that DAPI binds most strongly to AT rich regions of DNA, it is likely that the overall lower G+C composition of the zebrafish genome compared to mice and humans makes DAPI less effective for identifying regions such as pericentromeres that tend to be AT rich and to contribute heavily to chromocenters. We do agree with the reviewer that our statement may have been strong, given it is based only on EM data. We are now more precise in our claims, stating only that it is sufficient to accelerate establishment of condensed chromatin ultrastructure.

5. Figure 5. To what extent do ectopically gained H3K9me3 peaks in Smarca2 knockdown embryos overlap with endogenous peaks at 4.5 or 6.0 hpf?

Our direct chip data demonstrate that pericentromeres are a major site of H3K9me3 gain in Smarca2 knockdown embryos, and this target is clearly shared with 4.5 and 6.0 hpf samples. Additional analysis of overlap between ectopically gained peaks across the genome in morpholino injected embryos was more challenging due to the low amplitude of many peaks in morpholino injected embryos. Therefore, for this analysis, we chose to modify the stringency of our peak calling parameters to avoid inclusion of possible false positives. We found that as in 6 hpf wt samples, the bulk of these more stringent peaks (>90%) fell within annotated repeats. We then compared the similarity of enrichment profiles in various samples using the Diffbind software package. Applying a hierarchical clustering approach to the data reveled clustering of scramble morpholinos with 2.5 hpf samples whereas Smarca2 morpholino samples cluster with 4.5 and 6 hpf samples. This finding demonstrates that there is more similarity between peaks in Smarca2 MO injected embryos and later stages than between Smarca2MO injected embryos and controls. This finding, is now highlighted in the text, and in extended data Fig 7b**.**

6. Figure 3. Remove i) in the legend

We have removed i) from the legend

7. The authors could go further with this work, having uncovered a clear model in which heterochromatin establishment is established ectopically early, whether this affects development of the animal? So, for example to test development of the embryos after Smarca2-MO or PFI-3 injection. This will address the physiological relevance of the authors' finding of delayed heterochromatin establishment in the early zebrafish embryo.

We now include data describing developmental anomalies in both Smarca2 MOs and PFI-injected embryos in extended data 6.

REVIEWERS' COMMENTS:

Reviewer #1 (Remarks to the Author):

All my comments have been appropriately addressed.
Best regards, Nadine Vastenhouw

Reviewer #2 (Remarks to the Author):

I have reread the new draft from Goll and colleagues, and I am still intrigued. As reviewer 2, I am disappointed that the authors did not try to get the mutants to complement their morpholino studies. Does the Giraldez lab not have the necessary mutants?

Reviewer #3 (Remarks to the Author):

The authors have addressed all the points that I raised in my review. I support publication of this very nice work.

Reviewer 2 concern:

I have reread the new draft from Goll and colleagues, and I am still intrigued. As reviewer 2, I am disappointed that the authors did not try to get the mutants to complement their morpholino studies. Does the Giraldez lab not have the necessary mutants?

Rebuttal to reviewer 2:

We agree that testing H3K9me3 in maternal zygotic dicer mutants would have been a nice complement to morpholino studies. We did try to get these mutants, but after significant effort, could not identify any laboratory that could supply them. In order to obtain maternal zygotic *dicer* mutant embryos, we would need to cross adult females with *dicer* homozygous mutant germlines to homozygous or heterozygous *dice*r mutant males. However, because *dicer* is required for adult viability, such animals can only be obtained by transplantation the germline from homozygous mutant dicer embryos into wildtype embryos and rearing these animals to adulthood. Adult females created by this process are difficult to generate, have finite lifespans and cannot be maintained through breeding. Therefore, it is perhaps not surprising that we were unable to obtain find a laboratory that had these animals on hand.

Given that the dicer morpholino itself was only used as a secondary approach to support our finding that mir430 is required for heterochromatin establishment, and that we subsequently provide multiple, complementary lines of evidence demonstrating that degradation of Smarca2 (a known miR430 degradation target) underlies this requirement, we feel our combination of independent approaches meets the bar for rigorous analysis.
